# Graphene-Based Field-Effect Transistors in Biosensing and Neural Interfacing Applications: Recent Advances and Prospects

**DOI:** 10.1021/acs.analchem.2c03399

**Published:** 2023-01-24

**Authors:** Siva Kumar Krishnan, Nandini Nataraj, M. Meyyappan, Umapada Pal

**Affiliations:** † CONACYT-Instituto de Física, Benemérita Universidad Autónoma de Puebla, Apdo. Postal J-48, Puebla72570, Mexico; ‡ Department of Chemical Engineering and Biotechnology, National Taipei University of Technology, No.1, Section 3, Chung-Hsiao East Road, Taipei106, Taiwan; § Centre for Nanotechnology, Indian Institute of Technology, Guwahati781039, Assam, India; ∥ Instituto de Física, Benemérita Universidad Autónoma de Puebla, Apdo. Postal J-48, Puebla72570, Mexico

One of the most critical issues in the fields of health care, biomedicine, water quality monitoring, and food processing is the precise and label-free detection of biomolecules with high selectivity. Field-effect transistors (FETs) fabricated using two-dimensional (2D) channel materials have shown considerable promise, as the atomically thin channels allow for downsizing of transistors along with the enhancement of their sensitivity and selectivity in biomolecule detection. Specifically, graphene-based FETs (GFETs) have attracted tremendous attention because of their unusually high sensitivity, biocompatibility, and multiplexing capability, which allow them to detect multiple biomolecules as well as integrate with electrical readouts and digital microchips for point-of-care (POC) diagnostics. Here, we provide a complete overview of the recent progress in development and deployment of GFET biosensors for biosensing and neural interfacing applications. We examine the device parameters in depth, highlighting their significance in detecting a wide range of biomolecular targets, as well as the challenges and potential for incorporating them into various readouts for next-generation POC diagnostic tools. We also provide GFET biosensor design methodologies with a focus on recent advances in highly flexible and portable biosensor chips for POC handling. The interfacing of modern GFET devices with living biological systems such as flexible microtransistor arrays is highlighted. The use of GFET devices for *in vivo* and *in vitro* cell signal recording is discussed.

The revolution in biosensor technology during the past 20 years has been driven by the strong demand for biosensors in biomedical applications, especially for point of care (POC) diagnosis^1^ and healthcare monitoring.^2^ The biosensor market is predicted to grow at 8.9%, from 21.9 billion USD in 2019 to 36.6 billion in 2025.^3^ Indeed, biosensor technology has advanced enough for precise detection of a wide range of biomolecules, such as enzymes, bacteria, viruses, nucleic acids (NAs), and others, owing to the high demand and the advent of innovative nanomaterials.^4,5^ The development of highly wearable, portable biosensors has also received great interest in recent years.^4^ Such portable or wearable devices have long been desired for reliable monitoring of key biomedical and physiological information in a noninvasive or minimally invasive manner along with their applications in next-generation personalized healthcare systems, digital POC diagnosis, and healthcare monitoring.^5−7^ Moreover, the recent severe acute respiratory syndrome coronavirus 2 (SARS-CoV-2) outbreak, which created a historical global health crisis, has added urgency to developing fast and low-cost biosensors for rapid detection and POC applications. Although several diagnostic assays have already been developed using different detection platforms,^8,9^ the demand for low-cost, reliable, and portable digital electronic biosensors are in demand for faster screening.^10^

Field-effect transistor (FET)-based biosensors represent a unique class of analytical tools in healthcare monitoring for label-free, selective detection of chemical and biological species.^11^ They are adaptable to next-generation portable and on-site field-deployable sensing tools for precision on-site POC healthcare monitoring.^12^ Detection of biomolecules using FET biosensors involves structural and functional integration of recognizing moieties, such as NAs, proteins, enzymes, antibodies, aptamers, etc., with the active surface of the biosensor device, allowing label-free detection of analytes. Biorecognition events occur through selective binding of the biomolecular analyte of interest to the active surface of the biosensor (i.e., semiconducting channel), leading to a change in its local or interfacial potential or carrier concentration of the gate channel, producing current signals between the source and the drain (*I*_DS_) electrodes of an FET.^13^ The change in *I*_DS_ upon analyte binding can then be monitored by external hardware devices. Distinctive advantages of these FET-based biosensors over others are the possibility of signal amplification through external bias, label-free detection,^7^ unprecedented sensitivity, fast response, miniaturization, multiplexing, amenability for scale-up, and integration with signal processing electronics, in addition to their low-cost and the possibility of mass manufacturing.

Traditional FETs are fabricated on semiconductor materials such as silicon (Si)^14,15^ and III–V compounds (e.g., GaAs, GaN, ZnO, and In_2_O_3_).^16^ The rapid advancement of semiconductor technology has effectively doubled the number of components (e.g., number of transistors) in VLSI devices at a regular interval of time (every two years), following the trend predicted by Moore’s law.^17^ Although the semiconducting FETs have been scaled-down to sub-10 nm dimensions, exploration of alternative device geometry and new channel materials is still in progress for conventional applications of integrated circuits in computing and other areas. Such ultra miniaturization may not be needed in biosensors since hosting a drop of fluid would occupy a volume that does not need a submicrometer feature scale. One-dimensional (1D) Si nanostructures (e.g., nanowires and nanorods)^16,18^ and carbon nanotubes (CNTs)^19,20^ have been extensively studied for the fabrication of FETs because of their high charge carrier mobility and easy surface functionalization. However, the high cost and challenges associated with large-scale fabrication have impeded their use in biosensing platforms. Besides, precise control of the structure and electronic properties of these 1D materials remains a great challenge, as their complicated integration process often results in poor reproducibility of the fabricated devices.^21^ Alternatively, 2D materials consisting of atomically controlled thin crystalline layers have attracted increasing interest in the construction of FETs.^22−24^ The improved performance of these devices is associated with a host of unusual electrical properties of the 2D materials arising from their anisotropic geometry, flexibility, high mechanical strength, and high optical transparency, which have drawn much interest for their utilization as a channel material in FET devices.^25,26^ While all 2D materials have potential in FET-based sensing due to their reduced dimensions, well-defined bandgaps, and the possibility of high-density integration in planar devices, the carrier concentrations in graphene, graphene oxide (GO), and reduced graphene oxide (rGO) are still unbeaten.^27,28^ Thus, utilization of graphene and graphene derivatives is of tremendous interest for the development of biosensors, replacing conventional Si-based technology and exploiting their high electrical conductivity, superior carrier mobility, and high field velocity.^23,29−31^ Furthermore, the high mechanical flexibility, optical transparency, chemical inertness, and outstanding biocompatibility of graphene make it an ideal material for the development of next-generation POC diagnostic devices.^32^

The history of GFET biosensor development, from a simple graphene-based MOSFET device to wearable commercial digital biosensor chips and multiplexed mapping probes, is schematically depicted in Figure 1. The use of GFET biosensors has been diversified in different fields, paving the way for the development of modern POC diagnostic tools such as digital biosensor chips, highly flexible and wearable multiplexed biosensor devices useful for a wide range of biomedical applications, healthcare monitoring, and integration into brain tissues for accurate recording of brain functions. Specifically, since 2017, the advancement of GFET-based portable or chips is directed to commercialization from lab to market for real-world applications.

**Figure 1:**
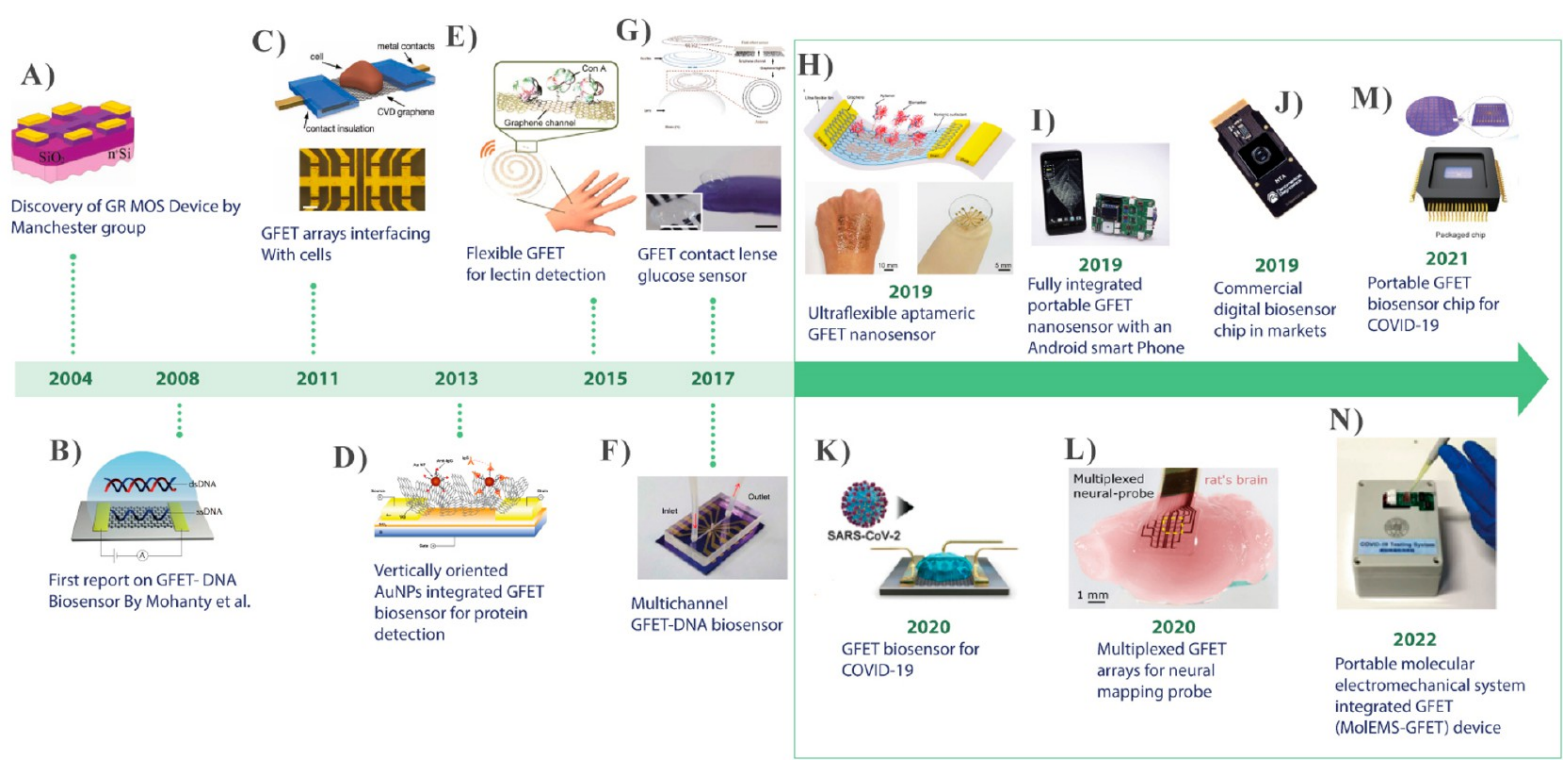
Chronological progress of GFET biosensor development and emergence of modern biosensors: (A) a graphene-based MOS device (Reproduced with permission from Novoselov, K. S.; Geim, A. K.; Morozov, S. V.; Jiang, D.; Zhang, Y.; et al. Electric Field Effect in Atomically Thin Carbon Films. *Science*. **2004**, *306* (5696), 666–669 (ref (34)). Copyright 2004 Science). (B) GFET-based DNA biosensor (Reproduced from Mohanty, N.; Berry, V. Graphene-Based Single-Bacterium Resolution Biodevice and DNA Transistor: Interfacing Graphene Derivatives with Nanoscale and Microscale Biocomponents. *Nano Lett*. **2008**, *8* (12), 4469–4476 (ref (35)). Copyright 2008 American Chemical Society). (C) GFET biosensor arrays interfaced with biogenic cells. (Reproduced from Graphene Transistor Arrays for Recording Action Potentials from Electrogenic Cells, Hess, L. H.; Jansen, M.; Maybeck, V.; Hauf, M. V.; Seifert, M.; Stutzmann, M.; Sharp, I. D.; Offenhäusser, A.; Garrido, J. A. *Adv*. *Mater*. Vol 23, issue 43 (ref (36)). Copyright 2011 Wiley). (D) A vertically oriented AuNPs-GFET biosensor used for protein detection (Reprinted by permission from Macmillan Publishers Ltd.: Nature, Mao, S.; Yu, K.; Chang, J.; Steeber, D. A.; Ocola, L. E.; Chen, J. *Sci*. *Rep*. **2013**, *3*, 33–36 (ref (37)). Copyright 2013). (E) Highly transparent, stretchable GFET used for lactin detection (Reproduced from Highly Transparent and Stretchable Field-Effect Transistor Sensors Using Graphene-Nanowire Hybrid Nanostructures. Kim, J.; Lee, M. S.; Jeon, S.; Kim, M.; Kim, S.; Kim, K.; Bien, F.; Hong, S. Y.; Park, J. U. *Adv*. *Mater*. Vol 27, Issue 21 (ref (38)). Copyright 2015 Wiley). (F) Real-time detection of DNA hybridization binding kenetics using multichannel GFET (Reprinted by permission from Macmillan Publishers Ltd.: Nature, Xu, S.; Zhan, J.; Man, B.; Jiang, S.; Yue, W.; Gao, S.; Guo, C.; Liu, H.; Li, Z.; Wang, J.; Zhou, Y. *Nat*. *Commun*. **2017**, *8*, 1–10 (ref (39)). Copyright 2017). (G) Wearable smart sensor integrated with contact lense for glucose monitoring in tears (Reprinted by permission from Macmillan Publishers Ltd.: Nature, Kim, J.; Kim, M.; Lee, M.; Kim, K.; Ji, S.; Kim, Y.; Park, J.; Na, K.; Bae, K.; Kim, H. K.; Bien, F.; Lee, C. Y.; Park, J., *Nat*. *Commun*. **2017**, *8*, 14997–15005 (ref (40)). Copyright 2017). (H) An ultraflexible aptameric GFET biosensor for biomarker detection (Reproduced from An Ultraflexible and Stretchable Aptameric Graphene Nanosensor for Biomarker Detection and Monitoring, Wang, Z.; Hao, Z.; Yu, S.; De Moraes, C. G.; Suh, L. H.; Zhao, X.; Lin, Q., *Adv*. *Funct*. *Mater*., Vol. 29, Issue 44 (ref (41)). Copyright 2019 Wiley). (I) A portable GFET biosensor integrated with an android smart phone for online detection of biomarkers (Reprinted from *Biosens. Bioelectron.*, Vol. *134*, Graphene-Based Fully Integrated Portable Nanosensing System for online Detection of Cytokine Biomarkers in Saliva, Hao, Z.; Pan, Y.; Shao, W.; Lin, Q.; Zhao, X., pp. 16–23 (ref (42)). Copyright 2019, with permission from Elsevier). (J) A typical commercial digital biosensor chip for biomarkers detection (Reprinted by permission from Macmillan Publishers Ltd.: Nature, Goldsmith, B. R.; Locascio, L.; Gao, Y.; Lerner, M.; Walker, A.; Lerner, J.; Kyaw, J.; Shue, A.; Afsahi, S.; Pan, D.; Nokes, J.; Barron, F. *Sci*. *Rep*. **2019**, *9*, 434–444 (ref (43)). Copyright 2019). (K) GFET-based antigen biosensor for COVID-19 virus detection (Reproduced from Seo, G.; Lee, G.; Kim, M. J.; Baek, S.-H.; Choi, M.; Ku, K. B.; Lee, C.-S.; Jun, S.; Park, D.; Kim, H. G.; Kim, S.-J.; Lee, J.-O.; Kim, B. T.; Park, E. C.; Kim, S. Rapid Detection of COVID-19 Causative Virus (SARS-CoV-2) in Human Nasopharyngeal Swab Specimens Using Field-Effect Transistor-Based Biosensor. *ACS Nano***2020**, *14*, 5135–5142 (ref (44)). Copyright 2020 American Chemical Society). (L) multiplexed GFET arrays as neural mapping probe (Reproduced from Garcia-Cortadella, R.; Schäfer, N.; Cisneros-Fernandez, J.; Ré, L.; Illa, X.; Schwesig, G.; Moya, A.; Santiago, S.; Guirado, G.; Villa, R.; Sirota, A.; Serra-Graells, F.; Garrido, J. A.; Guimerà-Brunet, A. Switchless Multiplexing of Graphene Active Sensor Arrays for Brain Mapping. *Nano Lett*. **2020**, *20* (5), 3528–3537 (ref (45)). Copyright 2020 American Chemical Society). (M) A portable GFET biosensor chip for detection of COVID-19 virus. (Reprinted by permission from Macmillan Publishers Ltd.: Nature Ke, G.; Su, D.; Li, Y.; Zhao, Y.; Wang, H.; Liu, W.; Li, M.; Yang, Z.; Xiao, F.; Yuan, Y.; Huang, F.; Mo, F.; Wang, P.; Guo, X. *Sci*. *China Mater*. **2021**, *64*, 739–747 (ref (46)). Copyright 2021). (N) A portable biosensor device based on molecular electromechanical system (MolEMS) integrated GFET (MoIEMS) for specific detection SARS-CoV-2 virus. (Reprinted by permission from Macmillan Publishers Ltd.: Nature, Wang, L.; Wang, X.; Wu, Y.; Guo, M.; Gu, C.; Dai, C.; Kong, D.; Wang, Y.; Zhang, C.; Qu, D.; Fan, C.; Xie, Y.; Zhu, Z.; Liu, Y.; Wei, D. *Nat*. *Biomed*. *Eng*. **2022**, *6* (3), 276–285 (ref (47)). Copyright 2022.)

Progress on the design of GFET biosensors and their performance in detecting diverse biomolecules has been reviewed in several articles.^16,26,28,29,33^ However, a comprehensive review covering the general aspects of GFET devices for detecting biomolecules of different characteristics, highlighting the critical aspects associated with their sensitivity and detection limit, is still lacking. Here, we present the state-of-art advances made in the fabrication of GFET biosensors, their performance in biomolecular detection, along with the key obstacles that must be overcome for large-scale utilization in healthcare monitoring. We also summarize the application and key challenges associated with the application of GFET microtransistor arrays for the intracellular recording of neuronal cell response, *in vitro*, and *in vivo* recording of brain activities.

## Structure and Function of FET and GFET

**Figure 2:**
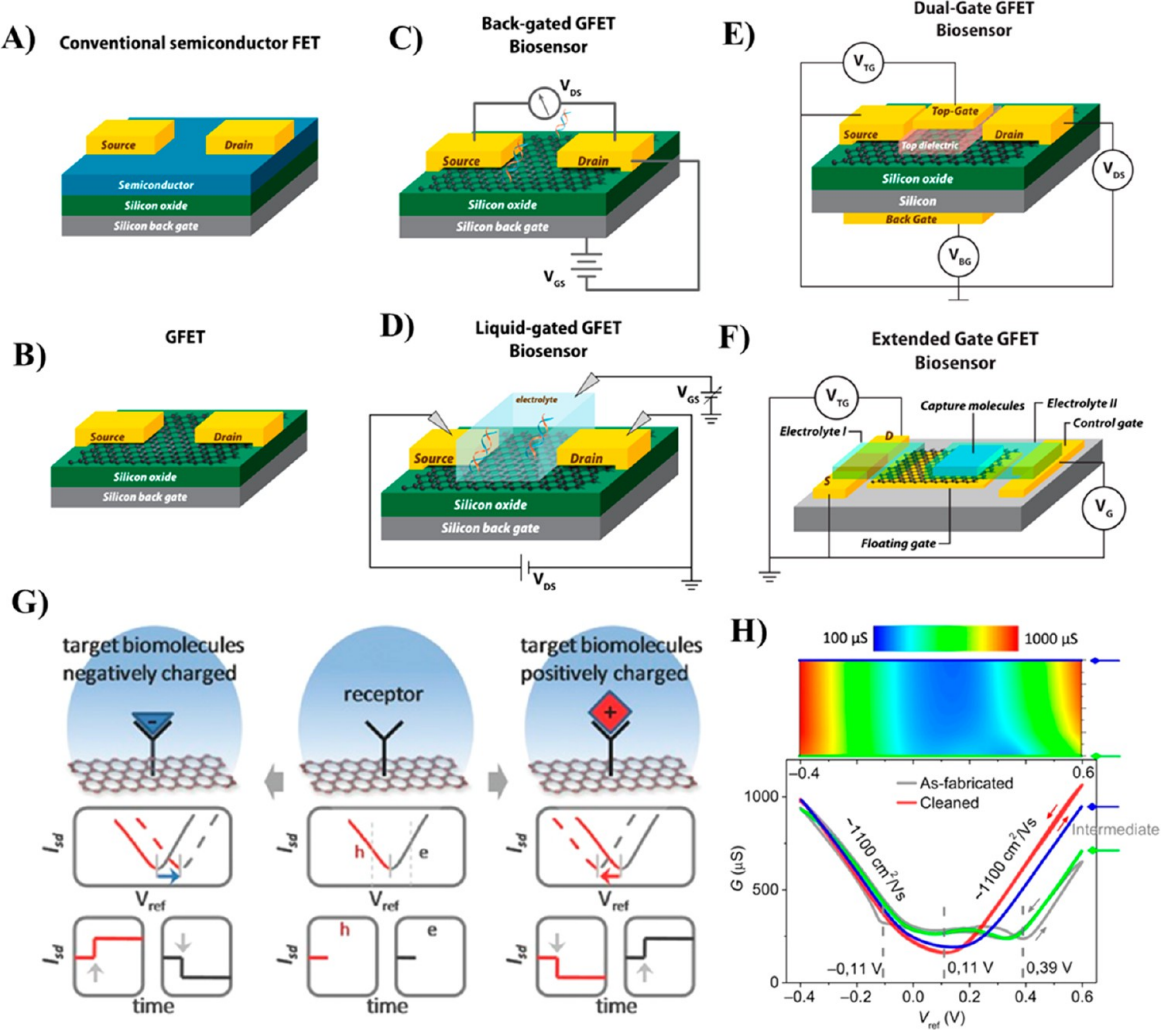
Schematic illustration of the structures of (A) conventional semiconducting FET, (B) GFET, (C) back-gated GFET, and (D) liquid-gated GFET, (E) dual-gate GFETs, and (F) extended (floating) gate GFETs configurations. (G) Pictorial presentation of sensing mechanism of liquid-gated GFET biosensors. The upper panel shows the type of receptor molecule binding on the graphene channel, and the lower panel displays the plots of *I*_SD_ vs *V*_ref_ and *I*_SD_*vs t*, respectively. Reproduced from Sensing at the Surface of Graphene Field-Effect Transistors, Fu, W.; Jiang, L.; van Geest, E. P.; Lima, L. M. C.; Schneider, G. F, *Adv*. *Mater*. Vol. 29, Issue 6 (ref (28)). Copyright 2017 Wiley. (H) Electrochemical cleaning of GFET. Upper panel shows the GFET sheet conductance mapping during electrochemical cleaning cycles under different applied liquid gate voltages (*V*_ref_) between −0.4 and 0.6 V. Lower panel presents the transfer curves of the GFET before electrochemical cleaning (gray line), during the 1st cycle (green line), 5th cycle (blue line), and after 10th cleaning (red line) cycles. Reproduced from Mayer, D.; Krause, H.-J.; Feng, L.; Panaitov, G.; Kireev, D.; Offenhäusser, A.; Fu, W. Biosensing near the neutrality point of graphene. *Sci*. *Adv*. *Adv*. **2017**, *3* (10), e1701247–e1701254 (ref (48)). Copyright 2017 American Association for the Advancement of Science.

A field-effect transistor (FET) is a three-terminal (*source*, *gate*, and *drain*) active device of high input impedance that uses an electric field to control the current flow. The semiconducting channel material is connected with the source and drain electrodes. The conductance of the semiconducting channel can be switched on and off by an applied voltage in the gate electrode (*V*_G_) that is electrostatically coupled through a thin dielectric layer (Figure 2A). The current flowing through the channel (the drain current, *I*_DS_) is tuned by an electric field perpendicular to the semiconducting channel, originating from the bias voltage applied between the gate and the source (*V*_GS_).^13^ The transverse electric field generated by the applied gate voltage (*V*_GS_) can either deplete the channel carriers resulting in no current flow between the source and the drain electrode (*off-state*) or increase the width of the channel, enhancing the current flow through it (*on-state*). Hence, the switching characteristics of the semiconductor FET device are dictated by the electrostatic coupling in the three-terminal devices which follow the one-dimensional Poisson equation:^24^
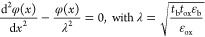
1where φ(*x*) is the potential distribution in the source–drain direction; λ is the transistor characteristics length; and *t*_b_, ε_b_, *t*_ox_, and ε_ox_ are the thickness and dielectric constant of the semiconductor channel and dielectric oxide layer (which is isolated from the gate electrode), respectively. Successful operation of a conventional FET device relies on its switching capability, defined as the ratio of device currents in these two states (*I*_on_/*I*_off_). An *I*_on_/*I*_off_ ratio >10^4^ is considered to be good for a conventional FET. The drain current, *I*_DS_, depends on the strength of the electric field on its mobile charge carriers, which can be expressed as
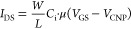
2where *C*_i_ is the capacitance of the gate insulator per unit area, μ is the charge carrier mobility in the channel,  is the width-to-length ratio of the channel, *V*_GS_ is the applied gate voltage, and *V*_CNP_ is the gate voltage at the charge neutrality point (CNP).

### Design and Operation of GFETs

GFET biosensors are ion-selective FETs (IS-FETs), which were first introduced by Piet Bergveld in 1970.^49^ The GFETs consist of a graphene channel, covered by an insulating layer such as SiO_2_ or Al_2_O_3_ to isolate the chemically reactive graphene channel from direct contact with ions and biomolecules, which enables a stable operation in electrolyte solutions (Figure 2B). GFET biosensors usually have a high signal-to-noise ratio due to the high carrier mobility and low electronic noise of graphene.^50^ Bonding of biomolecules to the surface of graphene channels can-effectively change the carrier density of graphene. Thus, the conductance of graphene can be sensitively modulated through the interaction with biomolecules. The Fermi level (*E*_F_) of the graphene layer can be shifted by applying a gate voltage (*V*_GS_) via reference electrode or due to the adsorption of biomolecules, thereby changing the conductance of the GFET device. For example, the conductance of *p*-type GO was seen to increase due to GO–DNA interaction by attaching a negatively charged single-stranded DNA (ssDNA) to the surface of graphene channels in GFET devices. The conductance was further increased by hybridizing ssDNA with its complementary DNA, which could be restored by removing the complementary DNA.^35^

Based on the mode of gate voltage application, the GFET biosensors can be divided into two major categories: (i) back-gated GFET (BG-GFET), and (ii) electrolyte-gated GFET (EG-GFET) biosensors, as schematically depicted in Figure 2C,D. A BG-GFET consists of metallic source and drain electrodes connected by a graphene conduction channel (Figure 2C). Metallic electrodes (e.g., 5 nm Cr/50 nm Au) are formed over the graphene channel to minimize contact resistance.^48^ In BG-GFET sensors, CVD-grown graphene is commonly transferred on highly conductive silicon substrates with a few atomic layered silicon dioxide insulating layers. The carrier density in the graphene channel can be modulated by applying back-gate voltage (*V*_GS_) through the highly conductive silicon substrate. In contrast, the EG-GFET geometry features a reference electrode together with the electrolyte functioning as the “gate electrode”.^51^ The semiconducting graphene channel and the gate electrode are in direct contact with the electrolyte solution, and the voltages *V*_G_ and *V*_D_ are applied at the gate and drain electrode, respectively (Figure 2D).^51^ The *V*_G_ and *V*_D_ are referenced to the source voltage, which is generally set to ground (i.e., *V*_S_ = 0). Also, the electrolyte-gate is coupled with the graphene channel through interfacial capacitance (*C*_I_).^52^ The graphene channel in the EG-GFET is in direct contact with electrolyte solution, which usually generates low leakage current between the gate and electrolyte solution.^53^ Highly stable additional passivation layers made of polyamide or epoxy are coated over the graphene channel to avoid contact between the source and drain through the electrolyte solution and hence to prevent any leakage current.^54^ This is highly important, especially when interfacing the EG-GFET arrays with neuronal cells to provide stable contacts with the neurons without disturbing the cell surface as well as to avoid leakage current in the electrolyte solution.^55^

Over the past few years, there have been many developments in GFET device design and configurations. For instance, construction of a dual-gated GFET configuration (Figure 2E) provides a promising way to enhance the sensitivity as well as a maximum on/off ratio, which is almost more than three orders magnitude higher.^56^ Meng et al.^57^ recently demonstrated such a dual-gated single-molecule GFET biosensor, where a single dinuclear ruthenium-diarylethene (Ru-DAE) complex, acting as the conducting channel, connected covalently with the nano-gapped graphene electrode. The utilization of high-K metal oxides (e.g., HfO_2_/Al_2_O_3_) plays a dominant role in achieving excellent performance of dual-gated FETs biosensors. In addition to the dual-gate GFETs, the extended gate (or floating gate) GFET configuration was also demonstrated (Figure 2F), in which there are two separate electrolyte regions (I and II) connected by a floating gate. The floating gate is capacitively coupled through the electrolytes to both the semiconductor channel (i.e., graphene) and the control gate.^51^ In this configuration, the base transducer is similar to the standard GFET (control gate), whereas the sensing element is formed by a specific functional layer on the extension of the metal gate as an external electrode that connects to the control gate as shown in Figure 2F. The capture molecules are immobilized over the floating gate in electrolyte region II, and the target molecules are detected in the electrolyte region II to generate the signal. This extended gate GFET has several advantages: it avoids direct contact with the target molecules with the semiconducting channel in region 1, higher stability and less drift, and less device-to-device variations in biological sensing.^58,59^

The working mechanism of EG-GFET is mainly the electrostatic interaction at the gate/electrolyte and electrolyte/channel interfaces depending on the type of ions binding at the surface of the graphene channel, which alter the electrical current in the GFET device due to the field-effect (Figure 2G). Based on the magnitude and polarity of the bias voltage applied to the gate electrode, the cations or anions from the electrolyte solution are moved toward the graphene channel. The ionic charges can increase or deplete the electronic charges present in the graphene channel, which give rise to the variation of the channel conductivity, along with a change in the drain–source current (*I*_DS_) flowing through the graphene channel.^51^ The charge carrier or conductivity of the graphene channel can be continuously shifted from the hole regime to the electron regime by operating the GFET from negative to positive bias. The minimum transconductance value of the graphene was observed at the transition point (0.11 V), which is known as the charge neutrality point, where the electron and hole densities are equal. Selective binding of negatively charged target biomolecules onto the graphene causes a positive shift of *I*_DS_ (*p*-doping), and binding of positively charged target molecules leads to a negative shift of *I*_DS_ (*n*-doping) due to the field-effect as shown in Figure 2G (lower panel). The lower panel of Figure 2G depicts the time-dependent current *I*_SD_ at a fixed reference potential *V*_ref_ (as indicated by the dashed gray lines). Upon binding a positively charged target molecule over the graphene surface, depletion of carriers (holes, indicated by “h”) occurs due to the field effect. As seen in the time-dependent *I*_SD_ at a fixed reference potential (*V*_ref_) depicted in Figure 2H (lower panel), the binding of positively charged molecules on the graphene channel causes a decrease of *I*_SD_ in the hole regime and an increase of *I*_SD_ in the electron regime, and *vice versa* for negatively charged molecules. However, binding of uncharged biomolecules on the graphene does not cause any change in the *I*_SD_, which signifies that the GFET biosensor does not show a response toward binding of uncharged biomolecules unless they induce a charge variation through a change in dipole moment between graphene and the substrate or through molecular interaction.

The modulation of *I*_SD_ at fixed *V*_ref_ in the graphene channel can be described in terms of the change in carrier density (Δ*n*), which is induced by and proportional to the total number *N* of charged biomolecules adsorbed on the graphene surface, through the relation
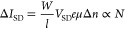
3where *W* and *l* are the width and length of the graphene channel, respectively; *e* and μ are the electronic charge and charge carrier mobility, respectively. As can be noted from eq 3, the biosensing performance of a GFET is directly proportional to the total number of biomolecules (*N*) conjugated to its graphene channel. Therefore, modification of surface immobilization density of targe molecules over the graphene channel is one of the key aspects for enhancing the sensitivity of GFET biosensors.

EG-GFETs exhibit multiple neutrality points and relatively high hysteresis with the variation of gate voltage, even with a pretreatment of SiO_2_ layer (used as the separator between the back gate and the graphene channel) with hexamethyldisilazane (HMDS) before the graphene-transfer process.^48^ This is because the presence of abundant surface contaminants in the SiO_2_ substrate leads to the generation of a large number of charged trap states at the graphene/electrolyte interface, despite baking the device at ∼200 °C and subsequent rinsing with isopropanol. The GFET is subjected to an *in situ* electrochemical cleaning process to remove the surface contaminants and obtain a stable neutrality point (Figure 2H, lower pannel). Each of the electrochemical cleaning cycles provides a distinct neutrality point in the transfer curves and suppresses the hysteresis. After the 10th cycle, the neutrality point of the GFET becomes highly stable, indicating the successful removal of surface contaminants. This is highly important to optimize the GFET to restore reliable characteristics, which significantly affect the sensing performance. Structural features and operational details of these two types of GEFT devices have been presented by Zhang et al.^28,33^

## Detection of Biomolecules Using GFETs

Diabetes is one of the most prevalent diseases, which affects millions of people worldwide.^60^ It is a chronic metabolic disease, which causes an abnormal increase of sugar levels in the blood. While it is not fully curable, early diagnosis and continuous monitoring are extremely effective for better control.^61^ After the first report of enzyme-immobilized electrodes for monitoring glucose by Clark and Lyons in 1962,^62^ substantial effort has been made to develop glucose monitoring systems utilizing numerous nanostructured materials.^63^ GFET-based biosensors have been developed and implemented for the sensitive detection of glucose levels in blood with excellent performance records.^64,65^ GFET channels are immobilized with a glucose-specific enzyme such as glucose oxidase (GOx), which functions as a recognition element during the sensing process.^66^

**Figure 3:**
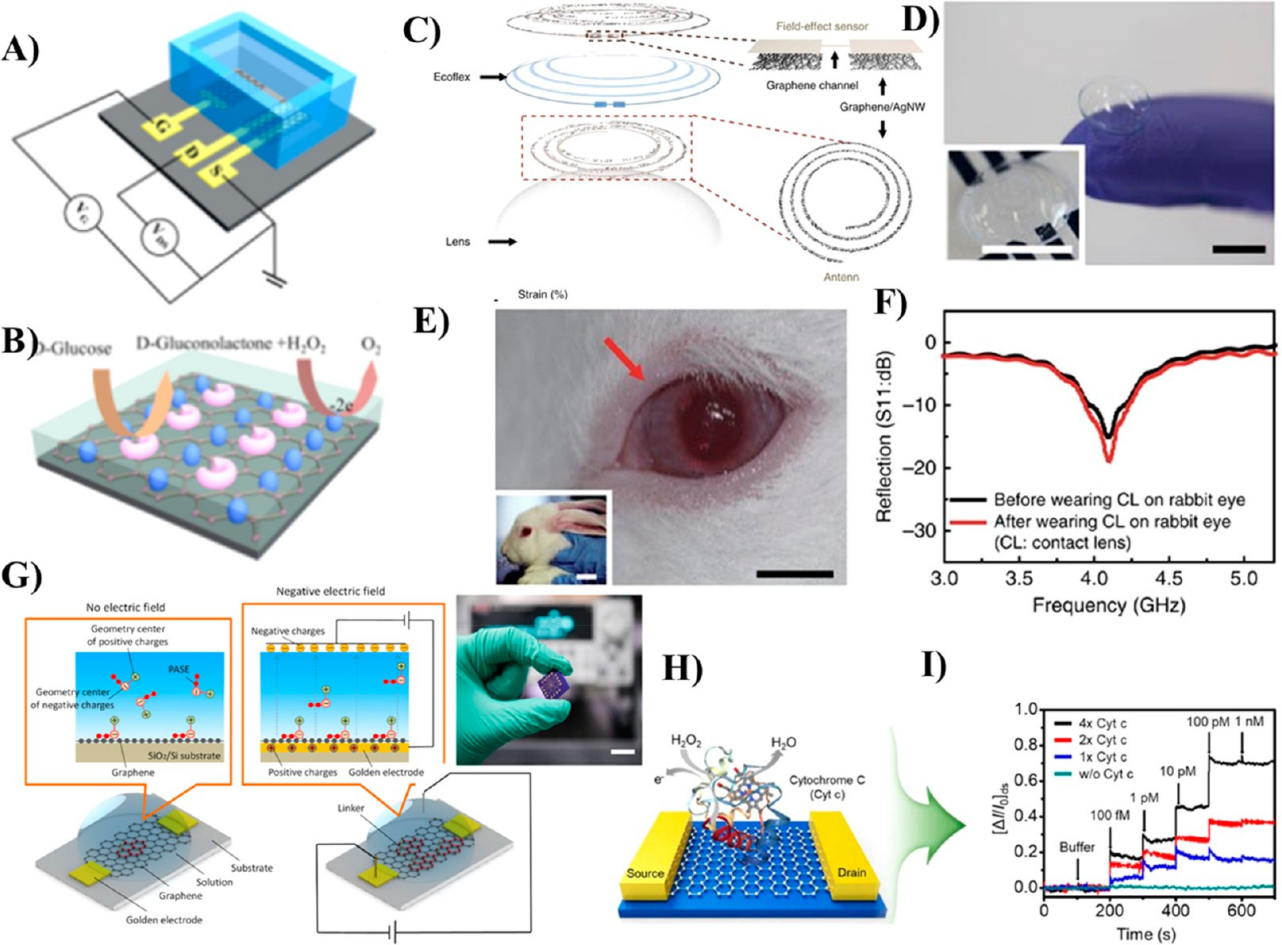
(A) Schematic diagram of a solution-gated GFET (EG-GFET)-based enzymatic glucose biosensor; (B) schematic illustration of GOx-catalyzed oxidation of glucose and oxidation of H_2_O_2_ cycles on the GOx-CHIT/Nafion/PtNPs/graphene gate electrode of an EG-GFET biosensor. Reprinted by permission from Macmillan Publishers Ltd.: Nature, Zhang, M.; Liao, C.; Mak, C. H.; You, P.; Mak, C. L.; Yan, F. *Sci*. *Rep*. **2015**, *5*, 1–6 (ref (68)). Copyright 2015. (C) Design of a wearable GFET based on enzyme-immobilized graphene/Ag nanowires integrated with a contact lens for monitoring glucose in tears and intraocular pressure; (D) photograph of a GFET-based transparent contact lens; (E and F) photograph of a wireless glucose sensor contact lens used for real-time continuous monitoring of glucose concentration in the eyeball of a live rabbit (Reprinted by permission from Macmillan Publishers Ltd.: Nature, Kim, J.; Kim, M.; Lee, M.; Kim, K.; Ji, S.; Kim, Y.; Park, J.; Na, K.; Bae, K.; Kim, H. K.; Bien, F.; Lee, C. Y.; Park, J. *Nat*. *Commun*. **2017**, *8*, 14997–15005 (ref (40)). Copyright 2017). (G) Schematic illustration of a GFET biosensor used for insulin detection. Right side image showing alignment of PBASE molecules immobilized over graphene channel upon applying the electric field is shown in the upper panel; photographic image of the fabricated GFET-insulin sensor device (scale bar: 1 cm) (Reproduced from Hao, Z.; Pan, Y.; Huang, C.; Wang, Z.; Lin, Q.; Zhao, X.; Liu, S. Modulating the Linker Immobilization Density on Aptameric Graphene Field Effect Transistors Using an Electric Field. *ACS Sensors***2020**, *5*, 8, 2503–2513 (ref (71)). Copyright 2020 American Chemical Society). (H and I) Schematic of the Cytochrome c (Cyt c)-modified GFET biosensor utilized for the detection of H_2_O_2_. Reprinted from *J*. *Ind*. *Eng*. *Chem*., Vol. 83, Park, J.; Kwon, O. S. Cytochrome C-Decorated Graphene Field-Effect Transistor for Highly Sensitive Hydrogen Peroxide Detection, pp. 29–34 (ref (72)). Copyright 2020, with permission from Elsevier.

Kwon et al.^67^ fabricated an enzymatic GFET glucose sensor utilizing a defective graphene layer as channel material and compared its performance with GFET sensors using pristine graphene and graphene mesh containing circular holes as channel materials. The GFET sensor fabricated with defective graphene initially exhibited a higher irreversible response to glucose due to strong chemisorption at edge defects. However, after GOx immobilization, the response irreversibility was substantially diminished, thereby reducing the sensitivity of the biosensor device. Their findings suggest that the graphene with edge defects can be used to replace linkers for immobilization of GOx with enhanced charge transfer across the GOx–graphene interface. GFET biosensors have been fabricated using metal nanoparticle (MNP)-grafted graphene as channel material and tested for glucose sensing. Zhang et al.^68^ reported the fabrication of highly sensitive glucose sensors based on EG-GFET with graphene gate electrodes modified with GOx (Figure 3A). The GOx enzyme immobilized on the gate electrode of the EG-GFETs can catalyze the oxidation of glucose in PBS solution and produce H_2_O_2_ near the gate electrode. The generated H_2_O_2_ oxidizes again by transferring electrons to the graphene gate electrode under a bias voltage (Figure 3B). The glucose detection was performed by monitoring the channel current, which is sensitive to the enzymatically generated H_2_O_2_ concentration. They also demonstrated that the sensitivity of the devices could be dramatically improved by modifying the graphene channel with platinum nanoparticles (PtNPs). The latter device exhibited improved glucose sensitivity with a LOD down to 0.5 μM, which is sensitive enough for noninvasive glucose detection in body fluids.

The development of transparent and stretchable electronic biosensor devices has gained substantial interest in recent years due to the potential advantages of wearable electronics and the growing demand for real-time health monitoring by noninvasive measurement of biomarkers in biofluids, such as in sweat, tears, salvia, and interstitial fluids.^2,69^ GFET-based transparent and stretchable smart contact lenses have been developed for wireless monitoring of glucose in tears and intraocular pressure (Figure 3C,D).^40^ For this purpose, silver nanowire integrated graphene (GR/AgNWs) composite was used as source and drain electrodes, and GOx immobilized graphene was used as an active sensing channel layer of the GFET device. The developed sensor could detect glucose with high sensitivity and selectivity in the presence of 50 μM ascorbic acid (AA), 10 mM lactate, and 10 mM uric acid (UA) with LOD of about 1 μM. Furthermore, the contact lenses were tested for *in vivo* monitoring of glucose in the eye of live rabbits during repeated eye-blinking and *in vitro* monitoring of ocular pressure in bovine eyeballs (Figure 3E,F). Although these smart lenses are capable of multiplexed sensing of both glucose and ocular pressure, simultaneous sensing of these functionalities was not assessed. Assessing biocompatibility and accurate monitoring in human subjects provides a great forward step in developing next-generation GFET biosensors for monitoring glucose in POC diagnosis.

The enzymatic GFET biosensors fabricated by immobilizing GOx enzymes over graphene often undergo degradation, apart from the challenges associated with their effective conjugation to promote electron transfer between GOx and graphene. Efforts have been made to develop nonenzymatic GFET biosensors of high stability using catalytic nanostructures as channel materials to overcome these challenges. For example, Ma et al.^70^ fabricated a GFET biosensor using AuNPs/rGO nanocomposite as channel material, which could detect glucose over a wide concentration range from 10 to 400 μM, with a detection limit (LOD) as low as 4 μM. The nonenzymatic solution-gated GFET device showed a high detection specificity toward glucose in the presence of other interfering species coexisting with human sweat, such as sodium chloride, urea, and lactic acid.

The glucose level in the human body is controlled by the secretion of insulin. Hence, precise monitoring of insulin levels in the body is of great importance for effective glucose regulation. GFETs have been utilized for highly sensitive and accurate insulin monitoring. For instance, Hao and co-workers^71^ reported an aptameric GFET biosensor for detecting interleukin-6 (IL-6) and insulin with sensitivity down to femtomolar concentrations (Figure 3G). Noncovalent immobilization of aptamers on graphene surface via PBASE linker molecules was used through π–π stacking between the pyrenyl groups of the linker and graphene planes. The densities of the PBASE linker and immobilized aptamer at the graphene surface could be effectively increased by applying an electric field during the immobilization process, thereby significantly enhancing the sensitivity. The sensor was tested for IL-6 and insulin sensing, in which the binding of IL-6 and insulin with aptamers induces a structural change in the aptamer from the folded state to a compact and stable formation and immobilizes onto the graphene surface. This structural change brings the charged aptamer and IL-6 linker close to the graphene surface, resulting in a noticeable change in the carrier concentration of graphene, affecting the *I*_DS_ of the GFET device. Application of gate voltage (*V*_G_) increases the density of the linker (PBASE) concentration over graphene surface. The sensor showed LODs of 1.22 and 1.66 pM for IL-6 and insulin, respectively, which could be enhanced further to 618 and 766 fM by applying an electric field of −0.3 V for 3 h during the PBASE immobilization process. The sensor could be utilized further to detect insulin in diluted human urine samples.^71^ This approach seems to be promising for enhancing the sensitivity through modulation of immobilized linker and aptamer densities on the graphene channel by an electric field.

GFET biosensors have also been widely employed for detecting H_2_O_2_.^74^ Lee et al.^75^ fabricated a Cytochrome c (Cyt c) protein functionalized graphene as channel material (Cyt c/GFET) for detecting H_2_O_2_ down to 10 fM concentration with a response time <1s (Figure 3H). Using glutaraldehyde as a cross-linker molecule, they functionalized the graphene channel surface with Cyt c through a Schiff-base reaction. The Cyt c molecules served as redox-active catalytic sites for the oxidation of H_2_O_2_ (magnified image in Figure 3H). A variation in *I*_DS_ was observed on interacting H_2_O_2_ with Cyt c, which increases the number of oxidized Cyt c^3+^ and decreases in the Cyt c^2+^ state. These changes modulate the charge-carrier density on the graphene channel surface and enable accurate detection of target H_2_O_2_ molecules. The fabricated biosensor revealed an excellent linear response to H_2_O_2_ between 100 fM and 100 pM concentrations. The sensing performance of the Cyt c-modified GFET was dependent on the concentration of Cyt c immobilized onto the graphene channel (Figure 3I). Functionalization of higher Cyt c protein concentration showed higher sensing performance for detection of H_2_O_2_.

Dopamine (DA) is one of the essential neurotransmitters released from nerve cells and renal, hormonal, and cardiovascular systems of the body and plays a vital role in the central nervous system as a neurotransmitter. DA dysfunction in human body’s nervous system causes severe neurological disorders, such as Parkinson’s disease,^76,77^ Alzheimer’s,^78^ schizophrenia, depression, and addiction.^79^ Therefore, accurate, rapid, and real-time monitoring of DA in a biological environment is of great importance for continuous monitoring and diagnosis of neurological disorders. Graphene and rGO-based FET biosensors have been successfully exploited to detect and monitor DA with exceptional sensitivity and selectivity.^80,81^ Liao et al.^82^ developed organic electrochemical transistors using graphene and rGO as channel materials for DA detection. The sensitivity could be enhanced by coating the graphene gate electrode with biocompatible polymers such as nafion or chitosan. The GFET fabricated using rGO was found to be capable of detecting DA in a wide concentration range of 5 nM to 1 mM with excellent selectivity under different interfering substances such as UA and AA. A needle-type GFET prepared with an rGO channel was also reported for sensitive dopamine detection.^81^ The GFET showed high sensitivity and good linear response in the 1 nm to 1 μM concentration range of DA and up to 500 μM concentration of AA.

Nonenzymatic detection of DA was also performed using flexible solution-gated organic GFET fabricated with Pt NPs-decorated rGO (Pt/rGO composite) as channel material.^83^ The Pt/rGO composite fabricated by chemical reduction of Pt ions over rGO surface was used as an active channel material. The source–drain electrode pattern was screen printed onto the Pt/rGO layer using polyaniline. Camphor sulfonic acid (PANI: CSA) was used as a substrate. This sensor could be utilized for real-time monitoring of DA with a response time <1 s, and the performance was strongly dependent on the concentration of Pt NPs over the rGO surface. The sensor exhibited high sensitivity with LOD ≈ 10^–16^ M concentration of DA and was highly selective in the presence of interfering molecules such as UA, AA, epinephrine (EP), and norepinephrine (NE).

Cortisol is a stress hormone, which is considered a major glucocorticoid released into the bloodstream by adrenal glands under physiological and/or emotional stress. Thus, accurate monitoring of cortisol levels is of great interest for controlling and preventing numerous stress-related ailments.^84^ A wearable, intelligent, and soft contact lens GFET biosensor was fabricated for wireless monitoring of cortisol in rabbit and human tears with smartphones.^73^ The immobilization of monoclonal antibody (c-mab) onto the ultraviolet ozone-pretreated CVD-grown monolayer graphene was performed via EDC [1-ethyl-3- (3-dimethyl aminopropyl) carbodiimide hydrochloride]/NHS (*N*-hydroxysulfosuccinimide) coupling reaction. Then, cortisol was accurately monitored upon its bonding with c-mab by monitoring the variation of the electrical signal in the GFET caused by the change in electrical conductance of the graphene channel. The biosensor could detect cortisol concentrations down to 10 pg/mL, i.e., with LOD much lower than the cortisol concentration in human tears. Moreover, a soft contact lens was fabricated by placing the sensor components (GFET sensor, capacitor, and resistor) over a stress-tunable rigid island-like hybrid support composed of photopatterned optical polymers and elastic parts made of silicone elastomers. The device was capable of *in vivo* real-time, wireless monitoring of cortisol in living rabbits and human tears sensibly via a mobile phone. This is a remarkable advancement in contact lens-based sensing platforms for noninvasive and mobile phone-based healthcare monitoring.

A wearable sensor based on platinum/GFET in an extended gate configuration (EG-GFET) was reported for real-time monitoring of cortisol hormone in biological fluids.^58^ The EG-GFET design consisted of immobilizing 61-base pair aptamers onto the Pt/single-layer graphene, which significantly overcomes the issues related to Debye screening and improves the device’s sensitivity. Also, a wearable 3D electronic chip was fabricated using EG-GFET through the CMOS process, which enabled real-time monitoring of cortisol in human sweat with high selectivity and negligible drift. The EG-GFET could detect cortisol in human sweat with LOD down to 0.2 nM concentration. The advancement sheds light on the possibility of integrating GFET sensors in miniaturized lab-on-chips for real-time monitoring. A flexible, portable, and disposable salivary cortisol sensor based on EG-GFET has been recently developed to detect cortisol in saliva samples.^85^ It was fabricated using direct ink-printing, where graphene ink was prepared and printed over a polyimide substrate. The device was functionalized with cortisol nucleic acid aptamer (3′-amino-modified oligonucleotide) through tetrakis(4-carboxyphenyl) porphyrin-linker. The sensor showed good sensitivity and selectivity toward cortisol for the concentration range of 0.01–10^4^ nM, which is lower than the cortisol concentration range (0.1–31.2 nM) in physiological saliva samples. Most importantly, the authors successfully integrated the EG-GFET sensor into a mobile phone for instantaneous and wireless detection of salivary cortisol, demonstrating the possibility of utilizing it for low-cost POC testing.

## GFET Biosensors for Nucleic Acid Detection

Nucleic acids (NAs) such as DNA (deoxyribonucleic acid) and RNA (ribonucleic acid) are polymeric biological molecules, consisting of nucleotide monomers. Each nucleotide contains three components: a five-carbon sugar, a phosphate group, and a nitrogenous base. The sugar is the deoxyribose in DNA, and in RNA, the sugar is the ribose. NA diagnostics has become one of the promising testing tools in modern medicine to analyze and treat infectious diseases.^86,87^ Therefore, identification and subsequent label-free and multiplexed detection of NAs are of great interest in personalized medicine,^88^ diagnostics,^89−91^ forensics,^92^ nanobioelectronics,^16^ and environmental monitoring.^93^ Traditional techniques employed for detecting nucleic acids are microarrays, isothermal amplification, and quantitative polymerase chain reaction (q-PCR).^94^ However, all these methods are pretty complex and expensive, as they need either prior signal amplification of the target genes or a complicated sample preparation process and signal detection. The GFETs have been extensively studied for NA detection owing to their atomic layer thick graphene channels, which can be readily functionalized with single-stranded probe DNA to detect specific target oligonucleotides with complementary sequences.^16,95−98^ Exploiting the advantages of GFETs, their sensitivity and selectivity were improved remarkably during the past few years; their LOD for NA detection has reached down to femtomolar (fM) or even attomolar (aM) concentrations.^99,100^

Ultrasensitive DNA detection by a GFET fabricated with rGO as channel material has been demonstrated by Cai et al.^101^ In this work, the authors introduced the concept of utilizing PNA (peptide nucleic acid) as a capture probe instead of DNA for targeting the complementary DNA sequence for the first time. The DNA detection was realized by the PNA–DNA hybridization event, which was monitored by a change in the electrical current response of the GFET. The rGO-FET biosensor modified with PNA exhibited high sensitivity with a LOD as low as 100 fM and high specificity to discriminate the complementary DNA from one-base mismatched and noncomplementary DNA. Ping et al.^102^ reported a scalable production of highly sensitive BG-GFETs functionalized with single-stranded probe DNA (ssDNA) of three different lengths (22-mer, 40-mer, and 60-mer) for label-free detection of target DNA. GFET biosensors showed a high affinity toward the target probe, and the detection sensitivity depended on the length of the target DNA strand. The LODs of the sensors were ∼100 pM for 22-mer target DNA, ∼100 fM for 40-mer target DNA, and ∼1 fM for 60-mer target DNA.

Another milestone accomplished in GFET biosensor technology is the fabrication of GFET arrays composed of multiple channels, which are extremely useful not only for the rapid and multiplexed analysis of biomolecule binding kinetics and affinities but also to improve the accessibility, specificity, and sensitivity of GFTE biosensors.^103,104^ The multiplexed GFET array platforms contain several devices within a single chip.^105^ The construction of such multiplexed GFET array platforms was reported by Xu et al.^39^ In this work, a DNA sensor was fabricated with graphene single-crystal patterned into multiple channels. The device was composed of six GFETs and utilized to analyze DNA hybridization kinetics with high sensitivity. The fabricated DNA biosensor could detect the binding kinetics of DNA hybridization upon the introduction of different concentrations of target DNA in each of the six channels. The sensor can detect target DNAs in their solutions in the 0.25–10 nM concentration range with a LOD of about 10 pM. Moreover, the biosensor can discriminate single-base mismatches in the target DNA sequence, demonstrating its potential for utilization in future diagnostic tools for reliable quantification of genetic variants. Mensah et al.^106^ reported label-free detection of DNA hybridization down to fM concentration, utilizing a GFET array. The CVD-grown graphene sheet transferred over Si wafer was lithographically patterned into 48 individual channels, and each channel was connected to a common source and an individual drain electrode (Ti/Au). The graphene channels of the GFET were covered with a thin (a few tens of nanometers) insulating layers of poly(l-lysine) (PLL) polymer, and the probe DNA oligonucleotide (20-mer) was electrostatically immobilized over it. The kinetics of the DNA hybridization process was monitored by detecting the shift in the Dirac point location during measurement. Hybridization of about 20 target DNAs could be detected using the fabricated GFET with high sensitivity and LOD up to 10 fM.

**Figure 4:**
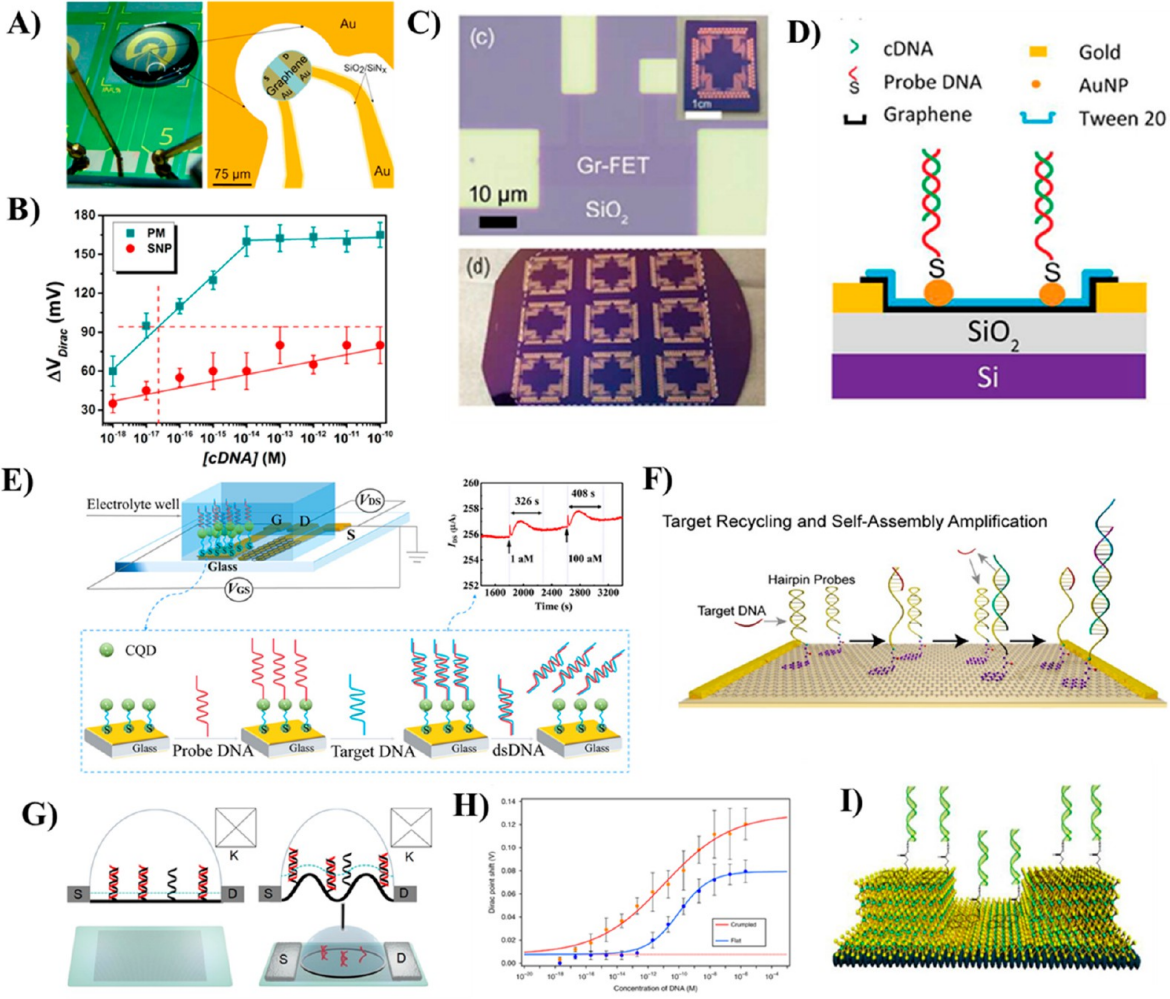
(A) Optical image of EG-GFET biosensor chip for label-free detection of DNA and (B) calibration curves for the GFET-based DNA sensor. Green squares refer to target DNA fully complementary to the probe and red circles to SNP target. The error bars are standard deviations of measurement with five different devices. Reproduced from Campos, R.; Borme, J.; Guerreiro, J. R.; Machado, G.; Cerqueira, M. F.; Petrovykh, D. Y.; Alpuim, P. Attomolar Label-Free Detection of Dna Hybridization with Electrolyte-Gated Graphene Field-Effect Transistors. *ACS Sensors***2019**, *4* (2), 286–293 (ref (99)). Copyright 2019 American Chemical Society. (C) Optical image of a GFET array biosensor device. The top-right inset shows an optical image of a Au-GFET biosensor chip. The bottom inset shows the optical image of an array of GFET chips in a 4 in. wafer. (D) Schematic of noncovalent binding of negatively charged complementary ssDNA molecules to PNA molecules on Au NP-supported graphene channel. Reproduced from Gao, Z.; Kang, H.; Naylor, C. H.; Streller, F.; Ducos, P.; Serrano, M. D.; Ping, J.; Zauberman, J.; Rajesh; Carpick, R. W.; Wang, Y. J.; Park, Y. W.; Luo, Z.; Ren, L.; Johnson, A. T. C. *ACS Appl*. *Mater*. *Interfaces***2016**, *8* (41), 27546–27552 (ref (111)). Copyright, 2020 American Chemical Society. (E) Configuration of the carbon quantum dot (CQD) functionalized SG-GFET for DNA detection. (Reproduced from Deng, M.; Li, J.; Xiao, B.; Ren, Z.; Li, Z.; Yu, H.; Li, J.; Wang, J.; Chen, Z.; Wang. Ultrasensitive Label-Free DNA Detection Based on Solution-Gated Graphene Transistors Functionalized with Carbon Quantum Dots. *Anal*. *Chem*. **2022**, *94* (7), 3320–3327 (ref (112)). Copyright 2022 American Chemical Society.) (F) Schematic of a GFET biosensor array, showing target recycling and triggered self-assembly amplification approaches for detecting DNA molecules at sub-fM concentrations. Reproduced from Gao, Z.; Xia, H.; Zauberman, J.; Tomaiuolo, M.; Ping, J.; Zhang, Q.; Ducos, P.; Ye, H.; Wang, S.; Yang, X.; Lubna, F.; Luo, Z.; Ren, L.; Johnson, A. T. C. Detection of Sub-FM DNA with Target Recycling and Self-Assembly Amplification on Graphene Field-Effect Biosensors. *Nano Lett*. **2018**, *18* (6), 3509–3515 (ref (100)). Copyright 2018 American Chemical Society. (G and H) Schematic illustration and characterization of flat and crumpled GFET used for DNA sensing. Reprinted by permission from Macmillan Publishers Ltd.: *Nat*. *Commun*. **2020**, *11* (1), 1543–1554 (ref (124)) copyright 2020. (I) Schematic illustration of a MoS_2_/graphene FET biosensor structure utilized for DNA detection by minimizing the Debye screening effect. Reprinted from *Biosens*. *Bioelectron*, Vol. 156, Chen, S.; Sun, Y.; Xia, Y.; Lv, K.; Man, B.; Yang, C., Donor Effect Dominated Molybdenum Disulfide/Graphene Nanostructure-Based Field-Effect Transistor for Ultrasensitive DNA Detection, pp 112128–112135 (ref (125)). Copyright 2020 with permission from Elsevier.

Campos et al.^99^ developed an EG-GFET-based DNA sensor device for label-free detection of target DNA with high specificity and ultrahigh sensitivity with LOD down to ∼25 aM concentration (Figure 4A). In this work, CVD-grown single-layer graphene was utilized as channel material. The target DNA was functionalized using PBASE-linker via π–π stacking interactions, followed by the passivation of the channel using ethanolamine (ETA) to prevent the nonspecific binding of the DNA probe molecules. Utilizing a large-area in-plane gate surrounding the graphene channel placed at its center (approximately 2500-fold bigger than the channel area) provided a uniform potential distribution inside the solution (uniform gating field). The EG-GFET DNA sensor showed excellent sensitivity for the perfectly matched target DNA molecules with a linear signal variation between 1 aM and 10 fM and a LOD of about 25 aM (Figure 4B). The LOD achieved in this work is one of the highest detection limits reported so far for DNA sensing using GFET biosensors.

Besides these advances, a few promising strategies have been developed for their implementation in POC detection platforms. As an example, a GFET-based DNA sensor was successfully integrated into a commercial PCB (printed circuit board), where the PCB served as the substrate, for rapid and sensitive quantification of target ssDNA.^107^ The graphene channel was formed by drop-casting graphene ink, and PNA probes were immobilized over it for the selective detection of ssDNA. The DNA sensor had a LOD of about 1 nM for complementary DNA. The Bio-GFET, fabricated through an inkjet-printing compatible manufacturing process, could detect target DNA within a few minutes with fewer amplification cycles. The work highlights the possibility of integrating GFET devices in POC diagnostic tools based on commercial PCBs.

A GFET-based DNA biosensor was constructed on an optic-fiber end by combining optical and electrical double read-out mechanisms for ssDNA detection.^108^ Two gold electrodes were prepared at the end of an optical fiber through laser etching to use as drain and source terminals. The GO layer was then coated at the surface of the fiber terminal to generate the GFET device. The binding of target ssDNA was performed by immobilizing fluorophore 60-carboxy fluorescein (60-FAM) aptamer on the GO channel surface. The sensing of the optical GFET is based on a dual mechanism: fluorescence resonance energy transfer (FRET) and electrical signal monitoring. Owing to the excellent optical quenching capability of GO, the fluorescence of the 60-FAM fluorophore immobilized onto the aptamer functionalized GFET could be efficiently quenched by GO through the FRET mechanism upon detection of target DNA, resulting in a change in the fluorescence intensity. Binding of target DNA leads to a change in the carrier mobility or conductivity of the graphene channel, which could be monitored by electrical read-outs. Therefore, both fluorescent intensity and electrical current could be simultaneously detected using a lab-made photoelectric double-channel detection system (PEDS). The utilization of such a novel approach enhanced the detection sensitivity of the Opt-GFET biosensor, achieving a LOD of about 10 nM concentration.

Various strategies have been adopted to enhance the sensitivity of GFET biosensors, which include the incorporation of metal nanoparticles,^109−111^ functionalization of graphene with carbon quantum dots (CQDs),^112^ detection near the neutrality point,^48^ and a combination of different signal amplification processes.^100,113^ Inorganic nanoparticles of different types can be used to functionalize the graphene surface of a GFET, which provides easy functionalization of receptor ligands and produces a large number of binding sites. Integration of metal nanoparticles such as Au and Pt with graphene has shown tremendous promise for enhancing their sensitivity in label-free biosensing. Metal NP incorporation in graphene channel enhances carrier mobility and biocompatibility and facilitates the immobilization of receptor molecules on the biosensor.^38,114^ Gao et al.^111^ reported a scalable fabrication process of Au NP-decorated GFET (AuNP-GFET) arrays for detecting nontarget DNA with high specificity (Figure 4C,D). On incorporating AuNPs over a graphene surface, the GFET device revealed improved carrier mobility of 3590 ± 710 cm^2^ V^–1^ s^–1^. The AuNP-GFET was readily functionalized with thiolated probe DNA molecules. The device exhibited excellent sensing capability with a detection limit down to 1 nM and high specificity against noncomplementary DNAs. Another study has shown that the functionalization of CQDs with the graphene channel surface in the SG-GFET configuration exhibits detection of DNA with much higher sensitivity down to 1 aM concentration (Figure 4E).^112^ In this work, the CQDs were immobilized on the graphene surface through mercaptoacetic acid to form the thiol group, and then the ssDNA probe was immobilized on CQD via π–π stacking interactions (lower panel in Figure 4E). Hybridization of target ssDNA with the ssDNA probe leads to the formation of dsDNA, resulting in the shift of *V*_Dirac_. Although they observed 2–5 order lower LOD values than the previous GFET biosensor for DNA detection, the developed sensor showed advantages such as a wide linear range (1 aM to 0.1 nM) and quick response time of about 326 s.

### Detection of Nucleic Acids near Neutrality Point of GFET

When a GFET is operated at the maximum transconductance point (i.e., neutrality point), where a small change in the gate voltage can induce a significant difference in *I*_DS_ current, it renders the highest sensing performance. However, the electronic noise is substantial at the point of maximum transconductance, which is considered an obstacle for achieving high sensitivity.^115,116^ The background noise of a GFET at the neutrality point of graphene can be reduced substantially as the electron density of states there is minimum.^48^ In addition, deposition of high-quality graphene layers over the dielectric substrate improves the charge carrier mobility remarkably, reaching up to 3800 cm^2^ V^–1^ s^–1^ in air.^117^ Therefore, the GFET operated near the neutrality point accomplishes maximum sensitivity with the highest signal-to-noise ratio. As has been demonstrated by Fu et al.,^48^ a GFET operating in ambipolar mode near its neutrality point can detect HIV-related DNA hybridization down to picomolar concentration with a maximum signal-to-noise ratio. The graphene surface of their GFET was first functionalized with a PNA aptamer, which binds with the target complementary HIV. Then, the PNA aptamer functionalized GFET was passivated with self-assembled Tween 20 to rule out possible false nonspecific positives. When operated near its neutrality point, the PNA functionalized GFET device rendered the best performance, enabling the detection of 11-mer ssDNA with a detection limit of 2 pM concentration.

### Signal Amplification Strategies

The sensitivity and LOD of GFET biosensors for DNA detection are limited by the binding affinity of the target oligonucleotide. Various signal amplification strategies have been developed and implemented for this, which are normally carried out at constant temperature.^87^ Maintaining constant temperature is accomplished using a system like a thermal cycler, which affects the amplification process. Alternatively, low-temperature isothermal amplification techniques have been widely employed to detect NAs.^118^ For instance, Han et al.^113^ developed an EG-GFET microchip adopting a microscale loop-mediated isothermal amplification (LAMP) strategy to detect viral DNA with high sensitivity. They used this strategy for real-time monitoring of the Lamda phage gene (LPG) as a proof of concept. The protons were released during the signal amplification steps, causing a gradual change in the Dirac point voltage. The sensor showed high sensitivity and LOD down to the fM range. Moreover, the device can generate an amplified signal within 16.5 min. Another promising signal amplification strategy based on target recycling and hybridization chain reaction (HCR) has been developed and implemented, which is capable of amplifying GFET current signal by several orders and enhancing its detection sensitivity. Gao et al.^100^ fabricated a GFET array with ∼20,000-fold improvement in sensitivity using engineered hairpin-structured probe DNA, which permits target recycling and hybridization chain reaction (HCR) to amplify the transduction signal (Figure 4F). In this work, instead of the commonly used ssDNA probe, the authors functionalized the graphene channel with hairpin-structured probe DNA (H1), which was exposed to a mixture of target DNA (T) and three helper DNAs (H2, H3, and H4).

The GFET experiences a positive shift in the Dirac voltage (p-doping effect of DNA binding) upon the exposure of complementary DNA, and the output signal was amplified by the HCR approach. The developed GFET array could detect the complementary DNA with high sensitivity and LOD down to 1fM using this HCR strategy. Furthermore, the GFET array demonstrated an excellent specificity toward single-base mismatches at the 3′ or 5′ end, indicating the possibility of its integration in POC diagnostic tools for sensible detection of DNA with high specificity.

### Strategies to Overcome Debye Screening in EG/LG-GFET Biosensors

It is well-known that the sensitivity and LOD of liquid-gated GFET biosensors are limited basically by the electrical double layer in ionic solutions, in which the intrinsic charge of an analyte is screened by the surrounding electrolyte ions, leading to a decrease in the gating response to recognition events (so-called Debye shielding).^119^ The extent of shielding, i.e., the effective sensing distance, is characterized by the Debye length (λ_D_), which determines how far from the sensor’s surface the analyte’s charge can be detected. In an aqueous solution, the λ_D_ is described by the equation^120^
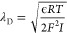
4where ε is the permittivity of the solution, *R* is the gas constant, *T* is the temperature, *F* is the Faraday constant, and *I* is the ionic strength of the aqueous solution/electrolyte. As shown in eq 4, λ_D_ is inversely proportional to the square root of ionic strength. The Debye length is typically about <1 nm in physiological solution with high ionic strength (∼150 × 10^–3^ M). Beyond the Debye length, the charges are electrically screened, resulting in only a small potential shift.^121^

To overcome the issues associated with Debye screening, poly(ethylene glycol) (PEG) coated on graphene surface was seen to be effective for increasing the screening length in solutions of high ionic strength and enhancing the sensitivity of the EG-GFET for detecting prostate-specific antigen (PSA).^122^ Additionally, co-immobilization of PEG- and PSA-specific aptamer over the graphene channel was seen to improve the specificity to detect PSA under physiological conditions. Piccinini et al.^123^ demonstrated one-order enhancement of λ_D_ magnitude and sensing range beyond λ_D_ by coating polyelectrolyte multilayers (PEMs) of opposite charges on the graphene surface of a GFET. They also proposed a theoretical model to describe the change of Debye length due to the variations of bulk ionic strength and polymer density. Their model confirms that the loss of entropy due to confinement of ions inside the PEM enables enlarging the λ_D_ and enhancing the sensitivity.

Few other promising attempts have been made in the design of GFET biosensors through modification of graphene channel architecture to enhance the Debye length. For instance, Hwang et al.^124^ demonstrated an approach to overcome the Debye shielding issue using a deformed (crumpled) graphene channel to construct a GFET biosensor for ultrasensitive detection of DNA and RNA molecules with LOD of the order of 600 zM and 20 aM in PBS buffer and human serum samples, respectively (Figure 4G). The Debye length fluctuates at the peaks and valleys of the crumpled graphene channel in comparison with the flat graphene, where it remains constant. The valley regions of the crumpled graphene expose more of the DNA; thus, the Debye length was effectively increased. Therefore, a weaker Debye screening results in a considerable enhancement in the sensitivity. DNA detection was realized first by the hybridization of immobilized (at the graphene channel) probe DNA and complementary DNA. The GFET fabricated using crumpled graphene showed a clear shift of Dirac point voltage (*V*_GS_) toward a more negative value with the increase of complementary DNA concentration (2 aM to 200 fM), whereas the GFET prepared with a flat graphene layer showed negligible Dirac point shift, as shown in Figure 4H. The hybridization tests were also performed using a PNA probe, which further enhanced the sensitivity of the GFET biosensor with LOD down to 600 zM for the detection of ∼18 molecules of DNA. Furthermore, the GFET could detect target miRNA (let-7b) spikes in undiluted human serum samples between 20 aM and 200 fM concentrations.

To prevent the noise generated by aqueous solution and the Debye shielding effect in EG-GFET, Chen et al.^125^ used a MoS_2_/graphene hybrid channel for ultrasensitive detection of DNA hybridization events (Figure 4I).The MoS_2_ layer coated over the graphene channel acts as a protective layer and induces polarization of DNA molecules and reduces the distance between DNA and the sensing surface (i.e., MoS_2_/graphene channel), increasing the sensitivity of detecting DNA hybridization events.^125^ While the MoS_2_ layer over the graphene channel could be formed by van der Waals interaction, the probe DNA molecules could be immobilized over it using PBASE linker molecules. The target DNA molecules were immobilized over the functionalized MoS_2_ surface via π–π stacking interaction. The MoS_2_-GFET could detect the DNA molecules over a broad concentration range, from 10 aM to 100 pM, and achieved the lowest LOD of 10 aM, which is the best LOD reported for DNA biosensors so far.

### Detection of Single-Nucleotide Polymorphisms (SNPs)

GFET-based biosensors have been developed for rapid and accurate discrimination of SNPs. For instance, Lal and co-workers^126^ developed a DNA strand displacement-based probe and utilized on LG-GFET biosensors for precise discrimination of single-mismatch DNA with high specificity. They designed double-stranded DNA (dsDNA) probes that attached onto the surface of the graphene channel for electrical detection of DNA strand displacement. The successful discrimination of a single mismatch over perfectly matched DNA targets was monitored by observing the change in the resistance of the graphene channel. For instance, immobilization of the DS probe onto the graphene channel increased the resistance from 40% to 60%. For the perfect match and single-mismatch DNA targets (10 μL concentration), the minimum resistance changes were observed to be about ∼84.9% and ∼46.0%, respectively. Using this technique, the developed GFET can discriminate the target DNA over a wide concentration range from 100 nM to 100 μM. The same research group also developed a miniaturized DNA-biosensor chip consisting of DNA-tweezers combined with GFET for wireless electrical detection of SNP down to pM concentration ranges.^127^ The combination of DNA-nanotweezers with GFET enabled achieving sensitivity about 1000-times higher than that reported for the detection of SNP previously. Specifically, they used a technique based on DNA strand displacement triggered by the target DNA that causes the strand displacement and opens the DNA nanotweezers on the chip. When the nanotweezers open and interact with target DNA, a strand displacement occurs, causing a charge difference. This process induces a change in resistance and shift of the Dirac point of the graphene channel of the GFET device. With the increase of target strand concentration, the DNA tweezers reveal the discrimination of a single mismatch. In addition, the wireless platform was established by connecting the GFET-biosensor chip with the wireless system using a microcontroller board, which allows the detection of electrical signals in a laptop or smartphone. This advancement of wireless and label-free discrimination of SNP with picomolar sensitivity is promising for the future diagnosis of genetic diseases, cancer, and other SNP-based alterations.

Aran and co-workers developed a digital GFET biosensor chip combining cluster regularly interspaced short palindromic repeats (CRISPR)-Cas9 with an LG-GFET device for quick and accurate discrimination of nontarget and target genes present in an intact genomic DNA sample without the requirement for amplification.^128^ The GFET combined CRISPR-Cas9 chip was constructed by immobilizing a catalytically deactivated Cas9 (dCas9) CRISPR complex (denoted as dRNP) onto a graphene channel in the GFET. The underlying detection mechanism is that the functionalized dRNP onto the graphene channel can selectively bind the target sequence in the genomic sample, which is complementary to the single-guide RNA (sgRNA) molecule within dRNP by unzipping the DNA double helix, rather than cleavage of reporter RNAs. The selective hybridization of target DNA with the complementary sgRNA in the dRNP complex modulates the conductivity of the GFET channel. A hand-held reader was integrated with the digital biosensor to detect the output signal for NA testing. The clinical application of the chip was also tested to detect gene mutations associated with Duchenne muscular dystrophy (DMD). The chip can detect two target sequences in DMD patients without the requirement of preamplification reactions with LOD of 1.7 fM concentration within 30 min, indicating a promising future for clinical applications.

The same research group also developed an SNP-digital GFET biosensor chip by integrating a CRISPR-Cas into SG-GFET, which can detect a single-nucleotide mutation in an unamplified DNA sequence without labeling or amplification.^129^ The graphene channel surface was functionalized with the CRISPR-Cas enzyme complexed with a target-specific guide RNA (gRNA) with a spacer of ∼20 nucleotides complementary to a specific DNA sequence. This Cas complexed gRNA interacts with specific DNA by recognizing protospacer-adjacent motifs (PAMs). When the RNA-guided Cas9 interacts with its PAM, it begins to unwind the DNA upstream of the PAM, and hybridization between the spacer sequence of the gRNA and the DNA target occurs, followed by cleavage of the DNA strand. Such recognizing events of target DNA by the RNA-guided Cas immobilized on the graphene channel create a local potential, resulting in detectable changes in the source–drain current of the GFET sensor, which can be measured in real time. Moreover, the SNP chip allows precise discrimination of single-nucleotide genomic mutations within homozygous and heterozygous DNA samples from patients with sickle cell disease within 40 min without the requirement of target amplification strategies.

### GFET Biosensors for RNA Detection

**Figure 5:**
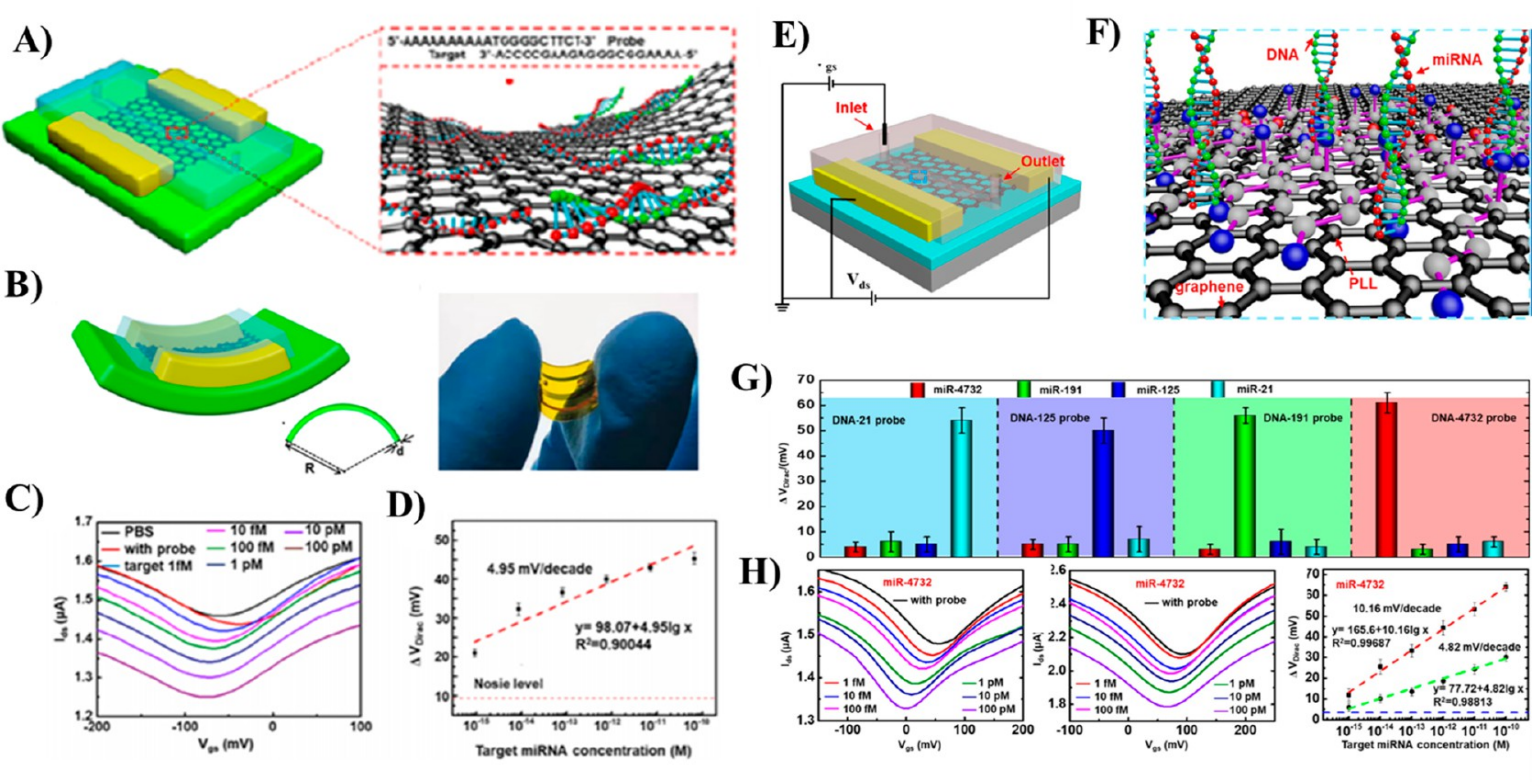
(A) Schematic illustration of a highly flexible SG-GFET biosensor device structure fabricated over PI substrate and utilized for miRNA detection. (B) The schematic structure of the flexible biosensor under bending and its optical image. (C) Transfer characteristics of the bent GFET biosensor under response to gate solution concentration with different target molecules and (D) corresponding calibration curve. Reproduced from Gao, J.; Gao, Y.; Han, Y.; Pang, J.; Wang, C.; Wang, Y.; Liu, H.; Zhang, Y.; Han, L. Ultrasensitive Label-Free MiRNA Sensing Based on a Flexible Graphene Field-Effect Transistor without Functionalization. *ACS Appl. Electron. Mater.***2020**, *2*, 1090–1098 (ref (135)). Copyright 2020 American Chemical Society. (E) Poly-l-Lysine-Modified GFET (P-GFETS) biosensor for breast cancer miRNAs detection and (F) schematic principles of GFET and PGFET for miRNA detection. (G) Sensing performance of the PGFET biosensor. (H) Transfer characteristics of PGFET biosensor at different miRNA concentrations. Reproduced from Gao, J.; Wang, C.; Wang, C.; Chu, Y.; Wang, S.; Sun, M. Y.; Ji, H.; Gao, Y.; Wang, Y.; Han, Y.; Song, F.; Liu, H.; Zhang, Y.; Han, L. Poly l-Lysine-Modified Graphene Field-Effect Transistor Biosensors for Ultrasensitive Breast Cancer MiRNAs and SARS-CoV-2 RNA Detection. *Anal. Chem.***2022**, *94*, 1626–1636 (ref (134)). Copyright 2022 American Chemical Society.

RNAs play an essential role in regulating diverse cellular processes such as the regulation of gene expression and genome maintenance,^130,131^ in particular, after the discovery of miRNA, which emerged as a new modality in medical diagnostics.^90^ Different research groups have demonstrated the utilization of GFET biosensors to detect miRNA with high sensitivity and specificity.^114,132,133^ Cai et al.^114^ fabricated a GFET by decorating its graphene channel with plasmonic Au NPs and utilized it for selective and label-free detection of miRNA. A simple drop-casting method was utilized to create an rGO–Au nanocomposite channel, and a peptide nucleic acid (PNA) probe was immobilized on the surface of Au NPs. The miRNA detection was realized at the surface of PNA immobilized Au NPs via PNA–miRNA hybridization. The device showed high sensitivity and the ability to accurately discriminate complementary miRNA from one-base mismatched miRNA and noncomplementary miRNA with LOD down to 10 fM concentration. Huang et al.^56^ enhanced the sensitivity of miRNA detection substantially using a dual gate SG-GFET based on GO/GR layered structure as active channel material. In this work, the GO/GR layered structure was generated by atomic layer oxidation of bilayer graphene, where the upper surface was oxidized to create GO and the bottom layer remained as graphene. The top GO layer enables the covalent conjugation of DNA probe molecules, and the bottom graphene layer functions as the signal transducer. The sensor could detect miRNA in the concentration range of 10 fM to 100 pM, and the sensitivity was about 1.75 times higher than that of the single-gate SG-GFET sensor due to the gate-controlled doping effect through the back gate. Gao et al.^135^ reported the development of a stable and flexible biosensor for ultrasensitive and specific detection of miRNA with LOD as low as 10 fM within 20 min (Figure 5A,B). The device was first fabricated on a flexible polyimide (PI) substrate and then integrated with a microfluidic chip containing an inlet and an outlet for sample loading and gate electrode placement in the liquid gate solution. The miRNA detection sensitivity of the flexible biosensor remained unchanged even after 35 bending cycles. This flexible sensor could detect complementary miRNA in the 1 fM to 100 pM concentration range with LOD down to 10 fM even after 35 mechanical bending cycles (Figure 5C,D). The work provided hope for developing flexible and wearable biosensor platforms for future POC diagnostics. Recently, a poly-l-lysine (PLL)-functionalized GFET biosensor was demonstrated to exhibit ultrasensitive detection of breast cancer miRNAs and viral RNAs (Figure 5E,F).^134^ The PLL was employed to functionalize the graphene channel to immobilize DNA probes through electrostatic interaction. The developed GFET biosensor showed high specificity and sensitivity for detection of complementary miRNAs between 1 fM and 100 pM concentrations (Figure 5G,H). The biosensor was also tested for detection of breast cancer miRNA and SARS-CoV-2 RNA in human serum and throat swab samples. The results showed excellent sensing performance for rapid and selective detection of miRNA and SARS-CoV-2 virus down to 1 fM concentration in 20 min. The developed sensor showed great potential for practical application in disease diagnostics and virus detection.

## GFET Biosensors for Protein Detection

Ultrasensitive detection of protein in living cells and physiological fluids is of great importance for the early diagnosis of cancer.^136^ GFET biosensors have been widely used for the sensitive detection of proteins.^16,137−139^ An EG-GFET biosensor was developed by co-immobilization of antibody fragment (F(ab′)_2_) and polyethylene glycol (PEG) on the graphene channel surface for sensitive detection of target protein thyroid-stimulating hormone (TSH) with LOD down to fM concentration.^140^ The device was constructed by surface functionalization of antibody fragments (anti TSH, F(ab′)2) and PEG on the surface of graphene via π–π stacking interaction. The presence of PEG at the surface of the graphene channel was found to reduce the Debye screening effect, which allowed the sensor to detect protein in both ionic buffers and blood serum. The results indicate that such electrolyte-gated GFETs are promising immunosensors suitable for POC applications.

Metal nanoparticle-supported graphene has also been implemented as active channel material for selective conjugation of target proteins.^139^ Adopting a novel approach of photocatalytic cleaning, Zhang et al.^141^ fabricated a renewable GFET for protein detection. The device could be reused by photocatalytically cleaning the protein molecules over the channel surface. For this purpose, they prepared rGO-encapsulated TiO_2_ composite (rGO@TiO_2_), which is highly photoactive, and used it to cover the rGO channel of a prefabricated FET. The sensor could detect D-Dimer with LOD as low as 10 pg/mL in PBS and 100 pg/mL in blood serum samples. The D-dimer molecules immobilized over the composite channel could be photocatalytically self-cleaned through UV-light irradiation to regenerate the biosensor. In this way, the TiO_2_-modified rGO-FET could be reused by immobilizing the D-dimer molecules several times. This innovative approach provides a viable solution for multiple uses of the same device and reduces the cost of protein detection. Apart from its excellent reusability, the sensor was able to detect different proteins in a single chip.

A digital chip biosensor (designated as Click-A+Chip by the authors) was developed by Sadlowski et al. utilizing multiple GFETs in one single chip for the precise detection of azido-nor-leucine (ANL) labeled proteins in parabiotic mice.^142^ The graphene channel was specifically immobilized with dibenzocyclooctyne-pyrene (DBOP) through click chemistry, which is capable of binding ANL-labeled proteins from one end, while the other end is conjugated on the graphene surface via π–π stacking. The binding of charged ANL-proteins on the graphene surface changes the conductivity of the channel, which could be monitored by a hand-held readout analyzer in the digital chip. Tests were performed with two types of ANL-proteins related to tissue rejuvenation such as Lif-1 and leptin in the parabiotic systemic milieu. The chip could significantly reduce the sample size, detection time, cost, and false positives and negatives. It offers promising opportunities for digital and portable platforms for future proteomic profiling and detecting the protein of interest.

## GFET Biosensors for Biomarker Detection

### Detection of Cancer Biomarkers

Biomarkers have emerged as potential diagnostic tools for cancer and many other diseases to define disease states precisely. The use of biomarkers for label-free detection of diseases provides a low-cost, rapid, specific, and highly sensitive POC diagnosis option.^143^ Sensitive detection of cancer biomarkers is essential for the early detection of the disease and their reliable early-stage prediction.^144,145^ An antibody-modified GFET biosensor was developed by Zhou et al.^146^ for sensitive detection of cancer biomarkers. The sensor was constructed by functionalizing anti carcinoembryonic antigen (Anti-CEA) onto single-layer graphene through noncovalent modifications using a PASE cross-linker molecule. The resulting anti-CEA-modified GFET sensor showed high specificity for detecting CEA protein with LOD below 100 pg/mL concentration. Mandal et al.^147^ developed a POC diagnostic tool based on a GFET biosensor with coplanar electrode configuration, integrated with a compact disc-like microfluidic system fabricated using four layers of poly(methyl methacrylate) (PMMA) for detecting PSA biomarkers. The sensor with optimized coplanar gate geometry enabled maximizing the capture of target PSA biomarkers and detected with a LOD of 1 pg/mL in blood serum and without any interference. Such coupling of GFET with a low-cost spinning disc-based microfluidic device platform is highly promising for practical implementation in POC diagnostics.

**Figure 6:**
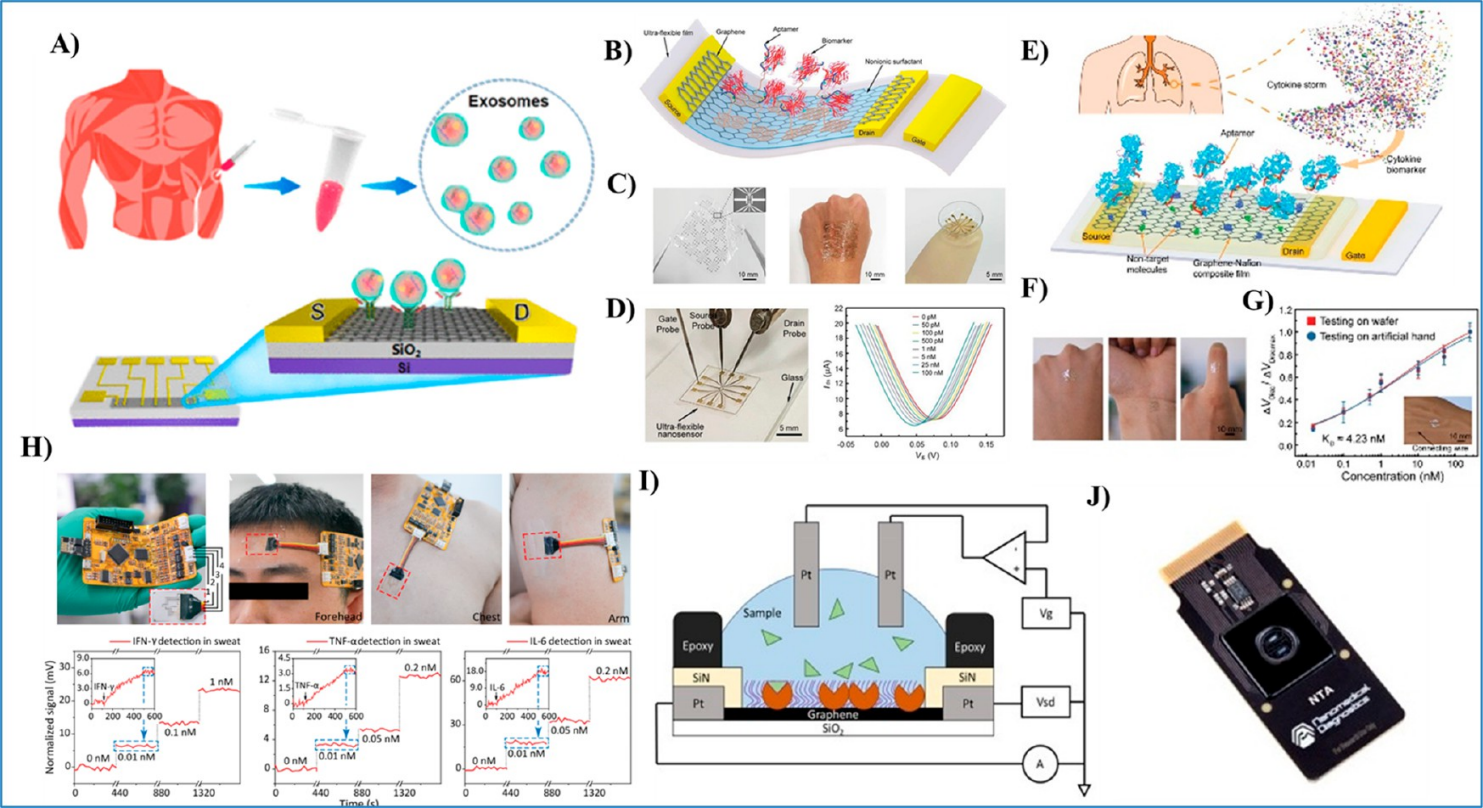
(A) Schematic illustration of a CD63 antibody functionalized rGO-FET biosensor utilized for label-free detection of exosomes in human blood. (Reproduced from Yu, Y.; Li, Y. T.; Jin, D.; Yang, F.; Wu, D.; Xiao, M. M.; Zhang, H.; Zhang, Z. Y.; Zhang, G. J. Electrical and Label-Free Quantification of Exosomes with a Reduced Graphene Oxide Field Effect Transistor Biosensor. *Anal. Chem.***2019**, *91*, 10679–10686 (ref (148)). Copyright 2019 American Chemical Society. (B) Schematic presentation of an ultraflexible aptameric GFET biosensor. (C) Photograph of a free-standing ultraflexible GFET biosensor array and an ultraflexible GFET sensor conformably mounted on a human hand, and a contact lens. (D) Photograph of the GFET biosensor placed on a glass slide for biomarker detection and transfer characteristic curves measured by exposing the biosensor to TNF-α solutions of different concentrations. (Reproduced from Wang, Z.; Hao, Z.; Yu, S.; De Moraes, C. G.; Suh, L. H.; Zhao, X.; Lin, Q. Ultraflexible and Stretchable Aptameric Graphene Nanosensor for Biomarker Detection and Monitoring. *Adv*. *Funct*. *Mater*. **2019**, Vol. 29 issue 52 (ref (41)). Copyright 2019 Wiley.) (E) An aptameric GFET biosensor used for cytokine biomarker detection. (F) Photographs of the flexible biosensor conformably mounted on a human hand and finger. (G) Normalized Dirac point shift Δ*V*_Dirac_, showing the sensing response of the flexible device placed on an artificial hand to the IFN-γ biomarker. (Reproduced from A Flexible and Regenerative Aptameric Graphene–Nafion Biosensor for Cytokine Storm Biomarker Monitoring in Undiluted Biofluids toward Wearable Applications., Wang, Z.; Hao, Z.; Wang, X.; Huang, C.; Lin, Q.; Zhao, X.; Pan, Y. *Adv*. *Funct*. *Mater*. **2020**, Vol. 31 issue 4 (ref (159)). Copyright 2020 Wiley). (H) The wearable and flexible GFET biosensor device fixed on different parts of the human body such as the forehead, chest, and arm for continuous monitoring of cytokine storm syndrome biomarkers. (Reproduced from An Intelligent Graphene-Based Biosensing Device for Cytokine Storm Syndrome Biomarkers Detection in Human Biofluids. Hao, Z.; Luo, Y.; Huang, C.; Wang, Z.; Song, G.; Pan, Y.; Zhao, X.; Liu, S. *Small***2021**, Vol. 17, issue 29 (ref (160)). Copyright 2021 Wiley). (I) Schematic of a commercial GFET biosensor chip structure. (J) Photographic image of the complete commercialized biosensor chip for monitoring biomarkers. (Reprinted by permission from Macmillan Publishers Ltd.: Nature, Goldsmith, B. R.; Locascio, L.; Gao, Y.; Lerner, M.; Walker, A.; Lerner, J.; Kyaw, J.; Shue, A.; Afsahi, S.; Pan, D.; Nokes, J.; Barron, F, *Sci*. *Rep*. **2019**, *9*, 434–444 (ref (43)). Copyright 2019.)

GFET biosensors have demonstrated their potential for the sensitive detection of cancer-related exosome biomarkers. For example, Yu et al.^148^ fabricated an FET biosensor chip using rGO as channel material to detect cancer-derived exomes selectively (Figure 6A). PASE was immobilized at the surface of the rGO channel through π–π stacking interactions between the pyrene group and the graphene surface. The antibody CD63 was covalently immobilized on the FET surface utilizing the interaction between the amino group of CD63 and the succinimide ester group of PASE. On capturing the exosomes by the specific antibody CD63, the net carrier density on the chip surface changed due to the contribution of the negative charges of the exosomes, resulting in a shift of the Dirac point. The negative Dirac point shift was well in accordance with the concentration of exosomes in the blood serum. The rGO-FET showed high sensitivity to cancer-derived exosomes with LOD down to 33 particles/μL. Moreover, the device was tested successfully for real-time sensing of exosomes in blood serum samples of healthy persons and prostate cancer patients.

To improve the sensitivity and capture efficiency of GFET biosensors, Ramadan et al.^149^ modified the graphene channel of their GFET with carbon dots (CDs) and tested it for exosome detection. The sensor was immobilized with primary CD63 antibodies using commonly employed PBASE linker molecules via π–π stacking interactions. In contrast to an rGO-FET, the Dirac point of the CDs-GFET was positively shifted upon binding of CD63 antibody at the channel surface. The CDs on the graphene surface modulate the electrical double layer and decrease Debye screening, leading to a two-order increase in sensitivity of the sensor and a three-order increase in the LOD compared to the corresponding values of unmodified GFET sensors. The CDs-GFET provided a LOD down to 100 particles/μL, offering the possibility of ultrasensitive detection of cancer-derived exosomes for early detection of the disease.

Metal nanoparticle-incorporated graphene nanocomposites have also been employed as channel material to improve the sensitivity for biomarker detection.^150^ Rajesh et al.^151^ fabricated a GFET array containing 52 GFETs utilizing antibody functionalized Pt NP decorated CVD-grown graphene and used for detecting breast cancer biomarker HER3 selectively. The prefabricated commercial Pt NPs were attached to the graphene channel via π–π stacking interaction using the bifunctional linker 1-methyl pyrene amine (PyNH_2_), where the pyrene moiety binds with graphene, whereas on the other end, the NH_2_ groups interact with Pt NPs. Then, the Pt NPs on the graphene channel were functionalized with HER3-specific genetically engineered thiol-containing single-chain variable fragment antibodies (scFv), which serve as bioreceptors for the target HER3 antigen. The authors modified the HER3 monoclonal antibody into scFv antibody containing a pair of cysteine residue(thiol), which facilitates the immobilization of the antibody onto the surface of Pt NPs embedded in the graphene channel. The device showed excellent sensitivity toward HER3 for its concentrations between 300 FG/mL and 300 ng/mL in PBS, with a LOD of 300 fg/mL. In addition, the sensor showed excellent specificity toward the control osteopontin solution of 30 ng/mL concentration. The results highlight the potential of GFETs for their utilization in the diagnosis of breast cancer, even in its early stage.

Aptamer-based GFET biosensors have also been developed and applied to detect thrombin biomarkers with high sensitivity.^152,153^ Thrombin is a biomarker for treating cancer cells, tumor growth, inflammation, etc.^154^ Yu et al.^153^ reported an aptamer-based SG-GFET biosensor for selective detection of thrombin biomarkers. They chose a thrombin-specific aptamer (ssDNA aptamer with 29 bases, Apt29:5′-SH-AGTCCGTGGTAGGGCAGGTTGGGGT-GACT-3′) for immobilization onto thiol modified gate electrode (Au electrode) to selectively recognize thrombin biomarkers. The thrombin molecule interacts with the ssDNA aptamers that are immobilized over the gate electrode, leading to stabilizing the thrombin (Cataion)-aptamer complex structure and inducing the folding of G-quadruplex structure aptamer molecules. When a gate voltage is applied to the SG-GFET, a capacitance of the electrical double layer (EDL) is generated at the interface of the gate/electrolyte and electrolyte/graphene interface. Thus, upon specific binding of thrombin molecules with the ssDNA aptamer-functionalized gate electrode, the capacitance of the EDL at gate/electrolyte changes, resulting in notable variation in the channel current (*I*_DS_). The sensor could detect thrombin biomarkers in the 1 fM to 10 nM concentration range with LOD down to 1 fM. Moreover, the response time of the detection was only about 150 s.

### Flexible and Stretchable GFETs for Biomarker Detection

Flexible and wearable GFET biosensors have gained substantial interest in recent years due to the possibilities of their utilization in continuous, real-time monitoring of disease-related biomarkers in biofluids like sweat, tears, saliva, and interstitial fluids.^155^ In fact, growing interest exists in developing flexible, wearable GFET biosensor arrays for the noninvasive monitoring of many important disease-related biomarkers.^156^ Kwon et al.^157^ reported one of the first works in this area and fabricated a flexible aptamer-based GFET biosensor using polypyrrole-covered CVD-grown nitrogen-doped few-layer graphene (PPY-NDFLG) for the detection of cancer biomarkers such as vascular endothelial growth factor (VEGF). The device was constructed by immobilizing an anti-VEGF RNA aptamer onto the PPy-NDFLG by modifying the side plane of PPy-NDFLG with glutaraldehyde-conjugated 1,5-diaminonaphthalene (DAN) through a Schiff-base reaction. In addition, the flexible biosensor was fabricated by supporting anti-VEGF RNA aptamer functionalized PPy-NDFLG FET onto a flexible and transparent polyethylene naphthalate (PEN) film. Upon specific binding of anti-VEGF aptamer with the target VEGF biomarker, a change occurs in the conductance of the PPy-NDFLG FET, leading to the recognition of target VEGF biomarker through an increase in *I*_DS_ with high sensitivity. The resultant aptasensor could detect VEGF between 100 fM and 10 nM concentrations within 1 s with an LOD of 100 fM. The authors demonstrated that the flexible aptasensor works even at a 3 mm bending radius and after multiple bending–relaxation cycles with only a <5% decrease in sensitivity.

Yang et al.^158^ reported a highly flexible GFET device with a high on/off ratio (∼1000) based on ultrafine graphene nanomesh (GNM), directly grown on a mesoporous silica template, utilized for detecting human epidermal growth factor receptor 2 (HER2) protein biomarker. In this work, the authors functionalized the GNM with HER-specific aptamer using PBASE as a linker to conjugate with the amino-modified HER2-specific aptamer by forming an amide bond for precise detection of HER2. The developed device is highly transparent and flexible and can be intimately attached to the human skin. The device can be bent and released continuously by folding and unfolding the motion of the wrist. A change in the charge-carrier density occurs on the surface of the GNM channel upon specific interaction of HER2 protein with the aptamer-modified GNM surface. The device showed a high binding affinity between the HER2 and aptamer and could detect HER2 in a wide concentration range (0.0001 to 10 ng/mL). The flexible device can also be utilized for real-time detection of breast cancer cells overexpressed with receptor 2 down to the single-cell level, highlighting its utility in next-generation low-cost clinical disease diagnosis.

Wang et al.^41^ reported an ultraflexible and highly stretchable GFET biosensor for the sensitive and reliable detection of liquid-borne biomarker TNF-α, an inflammatory cytokine closely related to fever in animation and inhibition of tumorigenesis (Figure 6B). The device was fabricated by depositing a monolayer graphene channel on thin Mylar film, and then immobilizing synthetic single-stranded DNA VR11 aptamer molecules that are specific to the target biomarker (TNF-α) onto the graphene channel via π–π stacking interaction. The ultraflexible device was tested at normal conditions and after mounting on a human hand and contact lens as displayed in Figure 6C. A lower thickness of the Mylar substrate of about 2.5 μm was used to deposit the graphene channel, and the sensor can be mounted on any surface that undergoes large bending, twisting, and stretching deformations (e.g., human tissue or skin). The binding of the biomarker on the aptamer-functionalized GFET channel induces a change in the carrier concentration of graphene, resulting in a change in the *V*_Dirac_ of the device. Increasing the concentration of TNF-α biomarker from 50 × 10^–12^ M to 100 × 10^–9^ M shifted the Dirac point (*V*_Dirac_) from 79 to 48 mV, suggesting an effective bonding between the aptamer and TNF-α. The change in *V*_Dirac_ with the variation of TNF-α concentration yielded an LOD as low as 5 × 10^–12^ M (Figure 6D). In addition, the specificity of the sensor was tested toward TNF-α and compared with the exposed control proteins (IFN-γ and IL-002 and bovine serum albumin (BSA)) at different concentrations under identical measurement conditions. The results demonstrated that the normalized Dirac point shift was about 5-fold higher than the control proteins at the same concentrations, suggesting that the sensor is highly specific to the target TNF-α biomarker. Moreover, the electrical properties of the biosensor were seen to be almost unchanged on bending, twisting, and stretching of the device.

The same research group fabricated a flexible and wearable aptameric GFET biosensor based on a graphene-Nafion nanocomposite as channel material for the sensitive detection of Cytokine biomarker (INF-γ) in undiluted human sweat (Figure 6E).^159^ The biomarker is closely related to inflammation, COVID-19, and cancer. Owing to its excellent flexibility and stability, the developed device was also mounted onto practically relevant supports such as artificial human hand and wrist to monitor INF-γ in human sweat (Figure 6F,G). The device exhibited excellent performance with LOD of about 880 fM concentration of INF-γ biomarker. The flexibility of the device was further tested by severely crumbling the device into a tiny ball from its original size. The sensor showed no detectable mechanical damage and exhibited only a 3.6% variation in its sensing performance even after 100 cycles of crumpling and decrumpling. All these results provide evidence for the incorporation of GFET biosensors into wearable POC diagnostic tools for rapid and convenient monitoring of disease biomarkers, including COVID-19 in human sweat by modifying the sensor with a probe specific to the biomarker.

A GFET-based intelligent and fully customized Android smartphone device was developed for the detection of cytokines biomarkers such as interferon (IFN), interleukin (IL), and tumor necrosis factor (TNF), which are closely related to COVID-19.^160^ This biosensor consists of 80 dual graphene channel FETs, functionalized with cytokine aptamer. Biomarkers could be selectively detected upon binding of charged cytokine molecules with the aptamers on the sensing graphene channel. Specifically, the aptamers bring the cytokine molecules close to graphene to form an electrical double layer (EDL) at the graphene and electrolyte interface, causing a modulation in charge distribution over the graphene–solution interface, altering the carrier mobility in graphene and causing a variation in drain–source current (*I*_DS_). The intelligent sensor could detect the biomarkers selectively within 7 min in various complex real samples such as human blood serum, saliva, and sweat with LOD of 476 × 10^–15^, 608 × 10^–15^, and 611 × 10^–15^ M concentrations of IFN-γ, IL-6, and TNF-α biomarkers, respectively. Importantly, this device was successfully integrated with an Android mobile phone, and data processing was realized with a Wi-fi module for on-site and self-detection of cytokines biomarkers in asymptomatic or mild COVID-19 symptomatic patients. Moreover, the device was fabricated on a highly flexible polyethylene terephthalate (PET) substrate and validated as a wearable sensor for consistently monitoring cytokines biomarkers in COVID-19 patients (Figure 6H). This demonstration is highly promising for POC applications, especially for continuous on-site monitoring of COVID-19 patients. However, the detection accuracy and large-scale fabrication cost of such biosensors are yet to be evaluated to assess the possibility of their POC implementation.

### Digital and Portable GFET Biosensor Chips for Biomarker Detection

The development of portable commercial chips for long-term digital monitoring of biomarkers has received tremendous attention recently due to the need for rapid diagnosis and real-time monitoring of different diseases. Integration of GFET biosensors with portable mobile phone platforms for accurate detection of cytokine biomarkers in saliva has been demonstrated.^42^ The system consisted of an aptameric GFET device with a buried-gate structure. It was fabricated by atomic layer deposition (ALD) of HfO_2_ (∼30 nm thick) film as gate dielectric layer over SiO_2_/Si substrate, on which a CVD-grown graphene layer was transferred through a wet floating-transfer technique. The graphene channel was functionalized with aptamer through PBASE linker molecules, and the sensor chip was mounted on a PCB board. Interleukin 6 (IL-6) was used to examine the sensing performance of the device by observing the structural changes of the functionalized aptamer upon interaction with IL-6. The structural change in the aptamer occurred due to the binding of the negatively charged IL-6 with the graphene surface, enabling the binding of aromatic amino acids in IL-6 with graphene via π–π stacking interaction. Such structure variation of the aptamer causes a change in the carrier concentration in the graphene channel and drain–source current (*I*_DS_). The sensor could detect IL-6 with LOD down to 12 pM within 400 s. In addition, the device exhibited excellent performance for real-time detection of IL-6 in human saliva solutions.

Commercial digital biosensor chips consisting of GFETs have been developed recently and are now available in the market for selective detection of biomarkers related to inflammation, autoimmune disease, and biomarkers such as human interleukin-6 (anti-IL6) and recombinant human IL-6 (IL6) in late-stage cancer patients (Figure 6I,J).^43^ In these devices, anti-IL6 is immobilized on a carboxyl functionalized graphene surface via standard carbodiimide cross-linker chemistry. The chips can monitor biomarkers more accurately than conventional assays such as colorimetric and nucleic acid-based PCR. The development of such GFET array chips is an important milestone in commercializing biosensor chips for real-world applications. A special type of chip based on GFETs (termed EV chip) was developed by Hajian et al.^161^ for label-free, rapid quantification of exosome biomarkers related to aging in plasma samples. This chip was designed with simple instrumentation, hand-held portability with a size of a large smartphone, less than 5 kg weight, and a low-cost electrical signal reader. The used biomarkers CD63 and CD151 are exosomes that carry specific biomolecules related to age and health. The CD63 antibody was functionalized on the surface of the graphene channel using PBASE linker molecules. Selective binding of the exosomes at the graphene surface changed its conductivity, causing a negative shift of the Dirac voltage (n-doping). The EV chip can accurately detect the exosomes from plasma with an LOD ≈ 2 × 10^4^ particles/mL. Moreover, the chip response could be accurately monitored by electronic devices such as laptops. The EV chip is suitable for commercial use, whether in a physician’s office or laboratories. Therefore, the chip can be utilized as a POC diagnostic tool to precisely monitor health and age-related diseases.

### GFET Integrated Microfluidic Platforms for Biomarker Detection

Microfluidic technology has shown great promise for portable, low-cost, and rapid quantitative detection in fluid samples such as suspended cells and particles in a small volume (in the range of μL to pL) without sample preparation steps for POC diagnostics.^162^ In particular, integration of the GFET biosensor with digital or droplet microfluidics has become a mature technology in the design of portable, single-platform, digital biosensing for sensitive detection of biomolecules and diagnosis of associated diseases.^147,163^ For example, Khan et al.^164^ fabricated a GFET-integrated portable microfluidic device for sensitive and real-time detection of thrombin biomarkers. The integrated device consisted of a microfluidic channel with an inlet and an outlet that traverses the source, drain, and an in-plane gate electrode. The measurements were performed under a fixed drain voltage (*V*_DS_), varying the *V*_GS_ voltage applied at the gate electrode. Upon binding of the thrombin biomarker at the graphene channel, the Dirac voltage shifted positively (toward the positive region) due to the positive charge of the thrombin aptamer, which makes the graphene surface p-doped. The device showed a remarkably improved sensing performance with LOD down to 2.6 pM concentration of thrombin in PBS solution.

## GFET Biosensors for Pathogenic Bacteria Detection

Bacteria are ubiquitous in the environment and although most are not harmful to humans, pathogenic bacteria are highly infectious and pose severe threats to public health.^165^ Pathogenic bacteria are responsible for water- and food-borne diseases, which pose a continuous threat to public health. World Health Organization (WHO) reports about 1–2 million human deaths caused by diarrhea.^166^ In particular, Gram-positive bacteria are the leading cause of a wide range of infections, regarded as the most common human pathogen associated with clinical diseases. The recent significant increase in the number and severity of bacterial infections requires rapid and efficient POC diagnostic tools for continuous monitoring of human health, environment, and food safety.^167^

GFET biosensors modified with antibodies have been frequently employed for bacterial detection with high sensitivity. Chang et al.^168^ developed an antibody-modified GFET using an rGO sheet as a semiconducting channel for sensitive detection of *E*.*coli* bacteria. The monolayers of GO sheets were selectively deposited onto the electrodes through a self-assembly process and subsequently annealed to convert them to rGO. The rGO-FET device was functionalized by immobilizing anti-*E*. *coli* antibodies over the rGO surface, which enabled sensitive and selective detection with LOD down to 10 CFU (colony-forming unit)/mL of *E. coli* cell concentration. Thakur et al.^169^ also reported an rGO-FET biosensor modified with *E*. *coli* antibodies (anti-*E*. *coli*) for selective detection of single *E*. *coli* bacteria. The detection was done by monitoring the change in the electrical conductivity upon binding with the negatively charged *E*. *coli*. To passivate the rGO channel, the FET was modified with a few nanometer-thick ALD-grown Al_2_O_3_ layer, which helped to avoid direct contact of water or unwanted species with the rGO channel surface, as well as to enhance the stability of the device. The device was able to detect *E*. *coli* in river water samples. Although the sensor could detect a single *E*. *coli* cell, some general issues such as antibody production and storage and transport difficulties currently limit practical application. To overcome these limitations, recent research efforts are directed to develop aptamers as sensing probes as they pose advantages such as facile modification, good stability, and high affinity toward various species from ions to whole cells.^170,171^

Utilizing pyrene-tagged DNA aptamer (PTDA) as sensing probe, Wu et al.^172^ fabricated GFET biosensors on Si chips for selective detection of *E*. *coli*. Each chip consisted of four single GFET devices. Functionalization of the DNA aptamer over graphene was accomplished through pyrene tag cross-linker molecules (pyrene phosphoramidite), which enabled a stable anchoring of aptamer onto graphene surface for specific detection of *E*. *coli* bacteria. The binding of *E*. *coli* on the GFET causes a conformational change of the aptamer, which brings the negatively charged *E*. *coli* close to the graphene channel surface. As a result, a significant right-shift (p-doping effect) in the transfer characteristic curves was observed. The aptamer-modified GFET device could detect *E*. *coli* bacteria down to 100 CFU mL^–1^ within a short time (∼ 72 s).

A high-performance portable graphene micropattern FET (GMFET) biosensor device combined with a microfluidic (MF) chip platform was developed for the early detection of Gram-positive and Gram-negative bacterias.^173^ The dual antibiotics functionalized GMFET (denoted as ABX-GM-FET) device involves two main layers. The top layer consists of a SIM card socket, a microcontroller, power supply, communication module, and electronic circuit. The bottom layer consists of a rechargeable battery. The device was integrated with a microfluidic chip placed in the SIM card socket in the top layer, and the inlet and outlet of the chip were connected with a syringe pump. The chip could selectively detect Gram-positive and Gram-negative bacteria in cultured samples in the 10^1^–10^3^ CFU/mL concentration range, offering a promising portable platform for real-time on-site detection of pathogenic bacteria in the environment.

## GFET Biosensors for Infectious Virus Detection

Diseases associated with viral infection pose one of the most significant public health challenges. These viruses generally originate from reservoir species such as mammals and transmit to humans to cause severe disease syndrome of different forms.^174^ Several virus-based diseases such as human immunodeficiency syndrome virus (HIV), severe acute respiratory syndrome (SARS), and the Middle East respiratory syndrome (MERS) coronaviruses have been appearing in various forms, causing outbreaks such as swine and avian influenza,^175^ Zika,^176^ Ebola,^177^ and most recently COVID-19.^178^ All these virus-based diseases have caused severe public health emergencies. Therefore, tracking and controlling the spread of these viruses are essential. In this regard, GFET-based electrical detection shows great promise for the rapid and accurate identification of these viral genome-based infectious diseases, as highlighted in the following subsections.

### GFETs for HIV Detection

HIV remains a major infectious species worldwide with no effective cure. Its severity is further complicated by opportunistic infections, especially in immune-compromised patients.^179,180^ Therefore, rapid, accurate, and early diagnosis of HIV and HIV-related diseases using portable diagnostic technologies is of great importance.^181,182^ Utilization of GFET biosensor combined with a microfluidic device for sensitive detection of HIV was first demonstrated by Kwon et al.^183^ in 2013. They fabricated a liquid-ion gated GFET device using graphene micropattern (GM) nanohybrids with close-packed carboxylated polypyrrole nanoparticle (CPPyNP) arrays as a flexible fluidic immunoassay, which enhanced the specific surface area of graphene micropatterned channels and provided stable sensing geometry in a liquid state. The immunosensor showed remarkable sensitivity for recognizing the target HIV biomarker with a concentration down to 1 pM. Kim et al.^184^ fabricated a GFET on flexible polyethylene terephthalate substrates for attomolar detection of an HIV-1 virus. Specifically, the probe molecules such as antibodies were decorated over the surface of the graphene gate using PBASE linker molecules. Upon dropping the virus solution, the Dirac point voltage shifted downward due to the electrostatic gating effect of graphene in the virus–antibody complex. The sensor could detect the HIV-1 virus with LOD down to 47.8 aM.

### GFETs for Ebola Virus Detection

**Figure 7:**
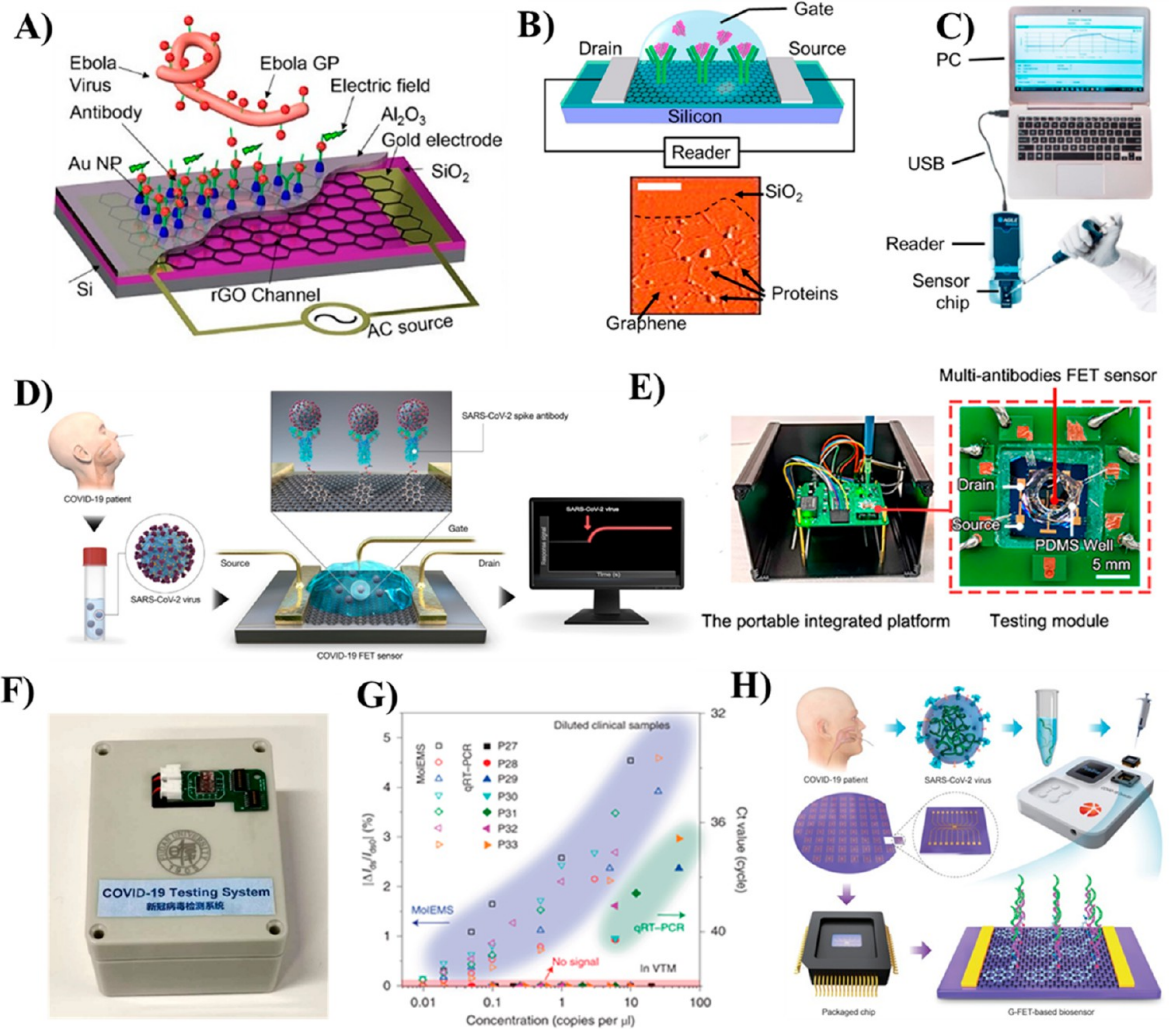
(A) Schematic diagrams of an insulator-gated GFET-based biosensorutilized for sensitive detection of Ebola virus using charge-injection–trapping–release–transfer mechanism at the channel–oxide and channel–electrode interfaces. (Reproduced from Maity, A.; Sui, X.; Jin, B.; Pu, H.; Bottum, K. J.; Huang, X.; Chang, J.; Zhou, G.; Lu, G.; Chen,J. Resonance-Frequency Modulation for Rapid, Point-of-Care Ebola-Glycoprotein Diagnosis with a Graphene-Based Field-Effect Biotransistor. *Anal*. *Chem*. **2018**, *90*, 14230–14238 (ref (186)). Copyright 2018 American Chemical Society.) (B) A GFET biosensor was utilized for detecting the Zika virus. Bottom: A typical AFM image of the graphene channel after successful protein attachment is presented at the bottom. (C) Schematic illustration of the completele GFET biosensor chip integrated with reader electronic platform consisting of a digital control, PC running control, and data processing software. Reprinted from *Biosens*. *Bioelectron*., Vol. 100, Afsahi, S.; Lerner, M. B.; Goldstein, J. M.; Lee, J.; Tang, X.; Bagarozzi, D. A.; Pan, D.; Locascio, L.; Walker, A.; Barron, F.; Goldsmith, B. R. Novel Graphene-Based Biosensor for Early Detection of Zika Virus Infection pp 85–88 (ref (187)). Copyright 2018, with permission from Elsevier. (D) A SG-GFET biosensor was utilized for the detection of SARS-CoV-2 virus from COVID-19 patients. (Reproduced from Seo, G.; Lee, G.; Kim, M. J.; Baek, S.-H.; Choi, M.; Ku, K. B.; Lee, C.-S.; Jun, S.; Park, D.; Kim, H. G.; Kim, S.-J.; Lee, J.-O.; Kim, B. T.; Park, E. C.; Kim, S. Rapid Detection of COVID-19 Causative Virus (SARS-CoV-2) in Human Nasopharyngeal Swab Specimens Using Field-Effect Transistor-Based Biosensor. *ACS Nano***2020**, *14*, 5135–5142 (ref (44)). Copyright 2020 American Chemical Society.) (E) Photographs of a portable integrated platform of multiantibody functionalized GFET biosensor for 10-in-1 COVID-19 antigen detection. The red dashed box indicates one packaged multiantibody FET sensor integrated into a printed circuit board (PCB). (Reproduced from Dai, C.; Guo, M.; Wu, Y.; Cao, B. P.; Wang, X.; Wu, Y.; Kang, H.; Kong, D.; Zhu, Z.; Ying, T.; Liu, Y.; Wei, D. Ultraprecise Antigen 10-in-1 Pool Testing by Multiantibodies Transistor Assay. *J*. *Am*. *Chem*. *Soc*. **2021**, *143*, 19794–19801 (ref (194)). Copyright 2021 American Chemical Society.) (F) Photographic image of portable MolEMS g-FETs biosensor chip for SARS-CoV-2 detection. (G) |Δ*I*_ds_/*I*_ds0_| responses and Ct values of MoIEMS-GFET in diluted clinical samples (∼P27–P33) in viral transport medium (VTM). (Reprinted by permission from Macmillan Publishers Ltd.: Nature, Wang, L.; Wang, X.; Wu, Y.; Guo, M.; Gu, C.; Dai, C.; Kong, D.; Wang, Y.; Zhang, C.; Qu, D.; Fan, C.; Xie, Y.; Zhu, Z.; Liu, Y.; Wei, D. *Nat*. *Biomed*. *Eng*. **2022**, *6*, 276–285 (ref (47)). Copyright 2022). (H) Portable GFET sensor system for on-site identification of COVID-19 positive patients. Bottom left: from Si wafer to plug-and-play GFET packaged chips. Top right: home-developed portable electrical detector. (Reprinted by permission from Macmillan Publishers Ltd.: Nature Ke, G.; Su, D.; Li, Y.; Zhao, Y.; Wang, H.; Liu, W.; Li, M.; Yang, Z.; Xiao, F.; Yuan, Y.; Huang, F.; Mo, F.; Wang, P.; Guo, X. *Sci*. *China Mater*. **2021**, *64*, 739–747 (ref (46)). Copyright 2021.)

The Ebola virus disease (EVD) was one of the most severe epidemic outbreaks in West Africa during 2013–2016, which was transmitted through over 28,599 people and caused more than 11,299 deaths.^177^ Chen et al.^185^ reported a GFET biosensor for real-time detection of Ebola glycoprotein (EPG) of the zaire strain with a detection limit down to 1 ng/mL concentration. The GFET was constructed with rGO as channel material, which was subsequently immobilized with an anti-Ebola antibody through Au NPs, and enabled capturing of the EPG antigens selectively. Effective conjugation of the Ebola antigen with the anti-Ebola immobilized antibody and subsequent change in the conductance of the rGO channel was monitored by observing the change in *I*_SD_ of the GFET. The sensor was capable of accurately detecting antigens in real samples such as 0.01× PBS/human serum/plasma samples, indicating its utility for rapid screening of EVD patients in early stages of the disease. Maity et al.^186^ developed an rGFET biosensor for sensitive and rapid detection (1–2 min) of Ebola glycoprotein antigen through an innovative resonance-frequency modulation technique. The detection was performed by exploiting antigen–antibody interaction and the charge transport inside the rGO channel or channel–electrode interface, i.e., a carrier-injection-trapping-release operation mechanism (Figure 7A). Because of the variation in the position of charge trapped inside the rGO channel/gate oxide (Al_2_O_3_) and channel–electrode interfaces, the traping-releasing time also changes at each charge trapping position. Such a variation in the charge trapping-releasing time can generate different relaxation frequencies corresponding to different trapping sites, which can be measured over a wide frequency range of the ac signal. Binding of Ebola antigen with antibody functionalized rGO channel generates the electric field on the gate oxide, which modulates the charge carrier concentration inside the channel. Utilizing this approach, the developed rGO-FET biosensor could detect Ebola glycoprotein antigen with LOD down to 0.001–3.401 mg/L at high and low frequencies, which is many orders higher than the limits of commonly utilized GFET biosensor devices.

### GFETs for Zika Virus Detection

The Zika virus is a mosquito-borne virus that originated in Uganda’s Zika forest in the mid-twentieth century. It is thought to be the cause of adult brain abnormalities and Guillain-Barre syndrome. While a nucleic acid test such as RT-PCR is the most common approach for Zika virus detection, GFET-based biosensors have also been used, particularly for detection at low concentration levels. Asahi et al.^187^ created a GFET device by covalently attaching monoclonal antibodies to the graphene channel surface, allowing for real-time, quantitative detection of natural Zika virus (ZIKV) antigens at low concentrations (Figure 7B,C). With LOD as low as 450 pM concentrations, the GFET biosensor showed outstanding responsiveness through capacitance change with the concentration of antigen (ZIKV NS1) in buffer solution, which is sufficient for clinical detection of the antigen. Furthermore, the biosensor detected Zika antigen and Japanese Encephalitis NS1 virus in simulated human serum and also in real samples with excellent specificity.

### GFET Biosensors for SARS-CoV-2 Detection

The SARS-CoV-2 virus is highly contagious and can spread rapidly. The virus causes severe respiratory distress,^188^ along with damage to different human organs such as lungs, heart, brain, kidney, and liver, and hence, its infection is life-threatening.^189^ Seo et al.^44^ were the first to use a GFET biosensor to detect the SARS-CoV-2 virus in clinical samples of human nasopharyngeal swab specimens using an antibody-functionalized graphene channel. The SARS-CoV-2 spike (S) antibody was used to functionalize the graphene channel of the GFET-based biosensor, which was then cross-linked with PBASE. The GFET was covered with PBS of pH 7.4 as an electrolyte to maintain the gating effect, as shown in Figure 7D. The spike (S) protein, which is a main transmembrane to the viral genome, was chosen for this purpose among the four structural proteins of SERS-CoV-2: spike (S), envelope (E), matrix (M), and nucleocapsid (N).^190^ The channel surface potential and the corresponding change in its electrical conductance were suppressed when spike protein was bound to the graphene channel surface, which was efficiently measured at different gate potentials. The LOD was 1 fg/mL in PBS and 100 pg/mL in clinical biological fluids using the SG-GFET device. The GFET was also able to detect SARS-CoV-2 spike protein in both cultured media (LOD: 1.6 × 101 pfu/mL) and clinical samples (LOD: 2.42 × 10^2^ copies/mL), confirming its ability to serve as a sensitive immunological diagnostic tool for detecting COVID-19, requiring no specific sample preparation or labeling.

Following the above work, many other groups developed GFET-based biosensing platforms for sensitive detection of the SARS-CoV-2 virus.^134,191^ Li et al.^192^ developed an AuNP-decorated GFET biosensor with a LOD of 2.29 fM concentration in throat swab samples and 3.39 fM concentration in human blood serum samples for fast detection of SARS-CoV-2 RNA within 2 min. The device was fabricated by immobilizing a phosphorodiamidate morpholino oligos (PMO) probe on the surface of AuNPs that were supported over a graphene channel. The RdRp gene was chosen as the target RNA gene sequence because it is involved in SARS-CoV-2 genome replication and transcription. Integrating plasmonic Au nanoparticles with graphene channel enabled high sensitivity and speedy detection because they exhibit excellent chemical stability and provide a greater surface-to-volume ratio and allow effective functionalization of PMO probe molecules at the surface. When the RdRp gene was hybridized with PMO-functionalized AuNPs, a significant shift in the biosensor’s Dirac point was noticed, which was analyzed to measure the concentration of the SERS-CoV-2 RdRp gene. The device detected the SARS-CoV-2 virus in clinically relevant real samples such as spiking serum and real clinical throat swab samples and distinguished between healthy and COVID-19 infected persons in real-time within 2 min.

Wei and colleagues^193^ fabricated GFETs with great sensitivity for detecting SARS-CoV-2 antibodies with LOD down to 2.6 aM. The antibody attaches to the S-proteins in the graphene channel, and biorecognition events occur when the antibody binds to the S-proteins. The conductance of the graphene channel changes noticeably as a result of the binding events. Clinical serum samples from COVID-19 patients were also used to test the GFET biosensor device. The sensors were able to identify COVID-positive patients (S1 to S9) with |Δ*I*_DS_/*I*_0_| ≥ 0.36% (Δ*I*_DS_ = *I*_DS_ – *I*_0_, where *I*_0_ is the initial *I*_DS_), which is more efficient than normal patients (N1–N9) with minimal |Δ*I*_DS_/*I*_0_| ≤ 0.1%). Even in samples diluted up to 50%, the sensors were able to detect SARS-CoV-2 antibodies with a LOD of 150 antibodies in 100 μL of serum in less than 2 min.

The same group also created a GFET biosensor with several antibodies on the graphene channel surface for sensitive detection of SERS-CoV-2 spike S1 protein in simulated saliva samples with LOD down to 3.5 × 10^–17^ g mL^–1^.^194^ Immobilization of several antibodies such as CR3022, n3021, and S1 improves antigen–antibody binding affinity in the recognition process, resulting in increased sensitivity and response time of the device. The biosensor showed outstanding sensitivity in clinical samples taken from nasopharyngeal swab specimens from COVID-19 patients and noncovid patients with an average diagnosis time of 38.9 s. Furthermore, they created a portable biosensor platform by accomplishing 10-in-1 COVID antigen pool testing with those multiantibody functionalized GFETs (Figure 7E). These portable multi-antibody GFET biosensors appear to be attractive platforms for developing POC diagnostic tools for COVID-19 patient screening in a large population. The developed portable device can precisely detect COVID-19 positive samples with a higher |Δ*I*_DS_/*I*_0_| value of 18.3% than the negative samples, indicating ideal antigen tests in clinical practice samples.

A variety of detection methods have been successfully integrated with GFET devices until now to increase the sensitivity and overall diagnostic time and avoid sample preparation steps. One of the promising approaches is combining nucleic acid assay with GFET platform for detecting SARS-CoV-2 nucleic acids.^195,196^ For instance, a direct nucleic acid assay using GFET functionalized with Y-shaped DNA dual probes was developed for the simultaneous detection of ORF1ab and N genes related to SARS-CoV-2 nucleic acid.^197^ The NA assay consists primarily of a Y-shaped DNA dual probe (Y-dual probe), which is functionalized onto the graphene channel surface through π–π stacking interaction with PASE cross-linker molecules. The functionalized Y-dual probe has a greater ORF1ab and N gene binding recognition ratio. As a result, the developed NA-based GFET showed high sensitivity (with a LOD of 0.03 copy μL^–1^) and fast response (nucleic acid testing in ∼1 min) toward SARS-CoV-2 nucleic acids. The developed Y-dual probe GFET-based NA assay demonstrated outstanding sensitivity in nasopharyngeal swab samples, even with trace amounts of SARS-CoV-2 virus (cycle threshold of 40.4) and a short diagnostic time of 40 s, which is up to 3 orders faster than existing NA-based assays. NA assay using an LG-GFET device immobilized with tetrahedral DNA nanostructures (TDNs) for highly sensitive direct detection of SARS-CoV-2 virus was also developed.^195^ The TDNs structure is formed by self-assembly by designed DNA sequences in 1× TM (Tris-HCl, MgCl_2_) buffer, which consists of a stiff tetrahedron base and flexible arm. The detection of the SARS-CoV-2 virus is mainly based on the electro-enrichment of suspended charged analyte at the gate electrode upon applying an electric field at the graphene liquid gate electrode due to electrophoretic transport. This testing enabled the detection of the SARS-CoV-2 virus with a fast response time of ∼80 s and high sensitivity (LOD close to 1–2 copies in 100 μL) in clinical saliva samples without the need for any additional NA extraction and amplification process. Importantly, the NA-integrated GFET biosensor assay avoids time-consuming nucleic acid extraction and PCR-based signal amplification tasks, and at the same time it exhibits sensitivity higher than the state-of-art detection methods such as PCR, CRISPER, and optical detection techniques, making it a potential platform for GFET-based NA diagnostic tools for future POC application, especially for quick testing of COVID-19 patients.

Another promising approach based on a molecular electromechanical system (MoIEMS) functionalized with LG-GFET (MoIEMS-gFETs) was developed for direct detection of SARS-CoV-2 RNA in nasopharyngeal swab samples.^47^ This device consists of a highly flexible and freely movable ss-DNA cantilever functionalized with an aptamer probe connected with a self-assembled stiff tetrahedral double-stranded DNA (ds-DNA) structure. Then, the MoIEMS is functionalized over the graphene channel in the LG-GFET. Upon selective recognition of target molecules, the change of the electrical potential of the graphene channel was monitored in real time. The sensor could detect the SARS-CoV-2 RNA in nasopharyngeal swab samples with LOD down to ∼0.02 copies per μL RNA in viral transport medium (VTM) with a detection time of approximately ∼0.1–4 min. A portable system was also presented using MoIEMS-gFETs, which is ideal for on-site detection in any place, including airports, clinics, and even at home (Figure 7F). The portable MoIEMS-gFETs device showed exceptional selectivity compared with the qRT–PCR standard tests (Figure 7G). Ke et al.^46^ developed a portable and fully integrated bifunctional GFET chip for simultaneous detection of SARS-CoV-2 RNA and IgG antibody protein (Figure 7H). The chip can detect down to ∼0.1 and ∼1 fg mL^–1^ in PBS for detecting SARS-CoV-2 RNA and IgG antibody protein, respectively. Additionally, the chip was validated by detecting in RNA extracts from the oropharyngeal swabs of ten COVID-19 patients and three healthy patients.

To increase the device-to-device reproducibility of the GFET biosensor, a remote floating gate (RGF) GFET configuration was also developed for reliable detection of SARS-CoV-2 spike proteins.^198^ The reported rGOFET sensor showed excellent sensitivity to detect SARS-CoV-2 in a saliva relevant sample with LOD down to a few pM concentrations. This extended floating gate configuration of rGOFET could potentially overcome the poisoning effect of FET biosensors in a clinical sample and increase the reproducibility of the biosensing devices. Another work by Bashir and co-workers^199^ developed a GFET utilizing crumpled graphene as channel material (named as CGFET) combined with reverse transcriptase loop-mediated isotherm amplification (RT-LAMP) technique to detect SARS-CoV-2 virus in clinical samples of viral transport media (VTM). Prior to the detection using the CGFET device, the RT-LAMP technique was utilized to amplify SARS-CoV-2 RNA in the N gene region from VTM clinical samples, resulting in the occurrence of primer consumption events during the amplification reaction. The developed CGFET device was tested in 20 clinical VTM samples (10 known positives and 10 known negatives) and could detect the SARS-CoV-2 virus in the 10 to 10^4^ copies/μL range in clinical samples conserved in VTM and successfully differentiate positive VTM from negative VTM in clinical samples within 35 min.

## GFET Biosensors for Metal Ion Detection

**Figure 8:**
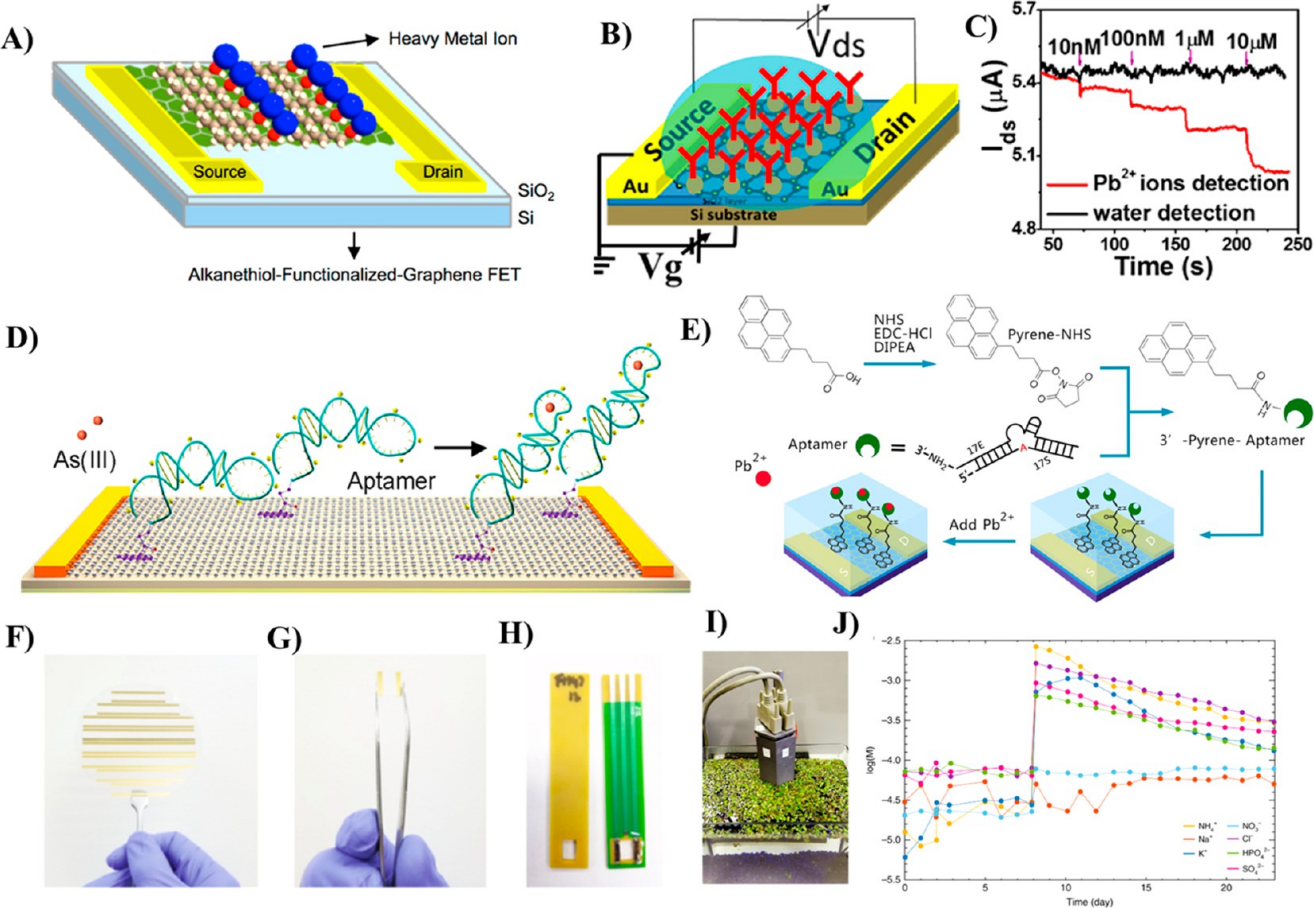
(A) Schematic illustration of an alkanethiol-functionalized GFET device used for heavy metal (Hg^2+^, Pb^2+^) detection. Reproduced from Afsharimani, N.; Uluutku, B.; Saygin, V.; Baykara, M. Z. Self-Assembled Molecular Films of Alkanethiols on Graphene for Heavy Metal Sensing. *J*. *Phys*. *Chem*. *C***2018**, *122* (1), 474–480 (ref (207)). Copyright 2018 American Chemical Society. (B) Schematic diagram of a DSH-functionalized AuNP-decorated rGO channel GFET device used for Pb^2+^ ion detection. (D) Real-time detection of Pb^2+^ in water using rGO/GSH-AuNPs-based GFET sensor. Reproduced from Zhou, G.; Chang, J.; Cui, S.; Pu, H.; Wen, Z.; Chen, J. Pulse-Driven Capacitive Lead Ion Detection with Reduced Graphene Oxide Field-Effect Transistor Integrated with an Analyzing Device for Rapid Water Quality Monitoring. *ACS Appl*. *Mater*. *Interfaces***2014**, *6* (21), 19235–19241 (ref (211)). Copyright 2014 American Chemical Society. (D) GEFT aptasensor arrays for detection of arsenite (Ar^3+^) ions. Reproduced from Li, J.; Tyagi, A.; Huang, T.; Liu, H.; Sun, H.; You, J.; Alam, M. M.; Li, X.; Gao, Z. Aptasensors Based on Graphene Field-Effect Transistors for Arsenite Detection. *ACS Appl*. *Nano Mater*. **2022**, *5*, 12848–12854 (ref (214)). Copyright 2014 American Chemical Society. (E) A portable GFET aptasensor capable of detecting Pb^2+^ ions in children’s blood. Reprinted by permission from Macmillan Publishers Ltd.: Nature, Treiber, Wang, C.; Cui, X.; Li, Y.; Li, H.; Huang, L.; Bi, J.; Luo, J.; Ma, L. Q.; Zhou, W.; Cao, Y.; Wang, B.; Miao, F. *Sci*. *Rep*. **2016**, *6*, 21711–21718 (ref (215)). Copyright 2016. (F) IS-FET fabrication process showing an optical image of 4 graphene wafer on the fused silica and parylene with gold contacts. (G) A single graphene device after being diced and ready to be mounted on a PCB. (H) Top and bottom view of IS-GFETs mounted on PCBs. (I and J) Real-time monitoring of ion concentrations using IS-GFET over a prolonged time. Reprinted by permission from Macmillan Publishers Ltd.: Nature, Fakih, I.; Durnan, O.; Mahvash, F.; Napal, I.; Centeno, A.; Zurutuza, A.; Yargeau, V.; Szkopek, T. *Nat*. *Commun*. **2020**, *11* (1), 3226–3238 (ref (217)). Copyright 2020.

Detection of toxic heavy metals such as Pb^2+^, Hg^2+^, Cu^2+^, etc. in aqueous solutions is of importance for human health and environmental safety.^200^ Numerous groups have developed GFET devices and implemented them for the sensitive detection of a wide range of metal ions including Hg^2+^,^201^ Pb^2+^,^202^ Cu^2+^,^203^ Fe^3+^,^204^ K^+^,^205^ Na+,^206^ Co^2+^,^206^ etc. As pristine graphene does not adsorb heavy metal ions selectively in most cases, the surface of the graphene channel in GFETs is functionalized with different functional groups to enhance their binding affinity and selectivity to heavy metal ions.^206,207^ Amine functionalization is commonly done to improve the selectivity of the devices by coating a monolayer of 1-octadecanethiol,^207^l-phenylalanine,^206^ or benzyl triethylammonium chloride (TEBAC)^208^ through noncovalent bonding strategies. Afsharimani et al.^207^ demonstrated that 1-octadecanethiol functionalized GFET can detect both Hg^2+^ and Pb^2+^ ions at 10 ppm level concentrations (Figure 8A)

Xiao et al.^209^ demonstrated that the modification of gate electrodes using glutathione (GSH) in an SG-GFET biosensor is highly effective for detecting Pb^2+^ ions. They modified the gate electrode (Au) with self-assembled GSH molecules over its surface via Au–S linkages, which serve as probe molecules for Pb^2+^ recognition. Poly(dimethylsiloxane) (PDMS) was used to seal the graphene channel and the gate electrode, and PBS solution was used as the electrolyte solution. Two electrical double layers (EDLs) were formed at the channel/electrolyte upon applying a gate voltage. The capacitance of the gate/electrolyte EDL changes upon selective binding of Pb^2+^ ions with GSH molecule, resulting in a Dirac point shift in the transfer curve of the SG-GFET. This sensor achieved high sensitivity with LOD reaching down to 10^–18^ M, quick response time of about 1 s, and selective detection in the presence of various interfering metal ions (Cr^2+^, Ca^2+^, Mg^2+^, K^+^, Co^2+^, Hg^2+^, Na^+^, Cu^2+^, and Fe^3+^).

To enhance the sensitivity and selectivity of the GFET ion-sensors, metallic NPs such as AuNPs were decorated with rGO and utilized as channel material to fabricate GFET sensors for the sensitive detection of Pb^2+^ ions.^210^ Chen and co-workers^210^ prepared a AuNP-decorated rGO channel utilizing AuNPs covered with a self-assembled monolayer of l-glutathione for selectively binding Pb^2+^ ions. The device could detect Pb^2+^ ions within 2 s in concentrations ranging between 10 nM and 10 μM. The LOD reached about 10 nM, which is lower than the maximum Pb^2+^ ion contamination level prescribed by the WHO in drinking water. The same research group reported a similar approach to fabricating an rGO-FET ion-sensor using a self-assembled rGO monolayer with a thick layer of Al_2_O_3_ as a passivation layer and immobilizing glutathione (GSH)-functionalized AuNPs over it for rapid detection of Pb^2+^ ions in water (Figure 8B).^211^ This sensor detected Pb^2+^ ions in water very quickly (within 2 s), with negligible signal drift and LOD of about 1 ppb in tap, lake, and river water with an accuracy of ∼75% (Figure 8C).

### Aptamer-Modified GFETs for Metal Ion Detection

Due to their extraordinarily high affinity and specificity for metal ions, DNA aptamers are another preferred material for altering the GFET surface. Aptamers are nucleic acids that have a high affinity and selectivity for binding to target molecules such as metal ions over a wide pH range. An et al.,^212^ made one of the first attempts at fabricating such aptamer-modified liquid ion gated flexible GFETs to detect Hg^2+^ ions in mussels selectively. Using glutaraldehyde (GA) as a bifunctional cross-linker molecule, the aptamer (3′ -amine-TTC TTT CTT CCC CTT GTT TGT-C10 carboxylic acid-5′) was noncovalently attached to the 1,5-diaminonaphthalene (DAN)-modified graphene surface. Upon binding of Hg^2+^ ions with the aptamer, the conductivity of the graphene channel changed, which was tracked by monitoring the change in the *I*_DS_ response. The flexible GFET-based aptasensor could detect Hg^2+^ ions in real-time with a LOD of 10 pM concentration. Although with reduced sensitivity, the aptasensor was able to detect nontargeted metal ions such as Cd^2+^, Co^2+^, Ni^2+^, Na^+^, Pb^2+^, Sr^2+^, Li^+^, and Zn^2+^ preferentially (smaller change of *I*_DS_). Single-stranded DNA (ssDNA) aptamer has been utilized for functionalizing graphene channels to fabricate an array of 6 × 6 GFETs on a single chip for the detection of Hg^2+^ ions.^213^ The aptamer was immobilized onto the surface of graphene channels in an SG-GFET array through two-step functionalization of cross-linker molecules. The aptasensor array demonstrated exceptional sensing capabilities for the detection of Hg^2+^ ions selectively in 100 pM to 100 nM concentration range within one second and with a LOD of 40 pM. Recently, Li et al.^214^ also demonstrated scalable GFET aptasensor arrays consisting of 100 GFETs for sensitive detection of arsenite (As^3+^) ions (Figure 8D). The aptamer functionalized GFET for detection of As^3+^ mainly relies on the conformationational change of the negatively charged aptamer upon interaction with As^3+^ ions. The developed sensor showed wide linear range from 0.05 to 1000 ppb and LOD of about 0.02 ppb.

Metal ions have also been monitored in real time using portable GFET-based aptasensors. Wang et al.,^215^ fabricated a portable GFET aptasensor for real-time detection of Pb^2+^ ions in children’s blood (Figure 8E). They chose 8–17 DNAzyme as the probe aptamer because it has an enzyme strand (17E) that cleaves the RNA base in the substrate (17 S) strand when Pb^2+^ ions bind to it. As a result, the graphene channel was functionalized with an 8–17 DNAzyme aptamer that took advantage of the π–π interaction between the pyrene cross-linker molecule at the 5′-end of 17E, which helped to avoid the nonspecific binding of Pb^2+^ ions and denaturation of 8–17 DNAzyme on the graphene surface. The detection of Pb^2+^ ions on the channel surface with 8–17 DNAzyme aptamer functionalized GFET is based on the replacement of RNA base adenine in 17S with DNA base (i.e., replacing the cleavable site ribonucleotide “A” with uncleavable deoxyribonucleotide “A”) in the 8–17 DNAzyme aptamer, which results in a significant change in *I*_DS_ of the device. This sensor was successfully used to detect Pb^2+^ ions in real blood samples from children with LOD less than 37.5 ng/L, which is substantially lower than the Pb^2+^ concentration safety standard for children’s blood. The study shows that GFET-based aptasensors can be integrated into POC diagnostic platforms for human health monitoring and disease diagnosis.

A unique technique based on single-atom enzyme functionalized GFET was reported for the sensitive and real-time monitoring of Hg^2+^ ions in Tris-HCl solution.^216^ The authors created a uniform dodecahedral-shaped N-doped carbon decorated with a single Fe site enzyme (Fe–N–C SAE) and inserted it into the gate electrode of an SG-GFET device that performed well for Hg^2+^ detection. The nitrogen (N) atoms on the Fe–N–C SAE selectively recognize Hg^2+^ ions by chelation between Hg^2+^ ion and N atom, while the catalytic site on the single-atom enzyme acts as a signal amplifier, allowing for the selective detection of Hg^2+^ ions. The addition of a single-atom catalyst significantly increased the sensitivity to Hg^2+^ ions, lowering the LOD to 1 nM in under 2 s. The findings clearly show that real-time detection for food safety and environmental monitoring applications is possible by utilizing such specially designed GFET sensors.

### Portable GFET Array-Based Detection of Metal Ions

GFET sensors have also been used to detect numerous metal ions simultaneously in real-time. Fakih et al.^217^ used large-area ion-sensitive GFETs (IS-GFETs) for real-time detection of various metal ions with sensitivity down to 10^–5^ M concentrations. They created a 54-GFET array mounted on a PCB board with two sides coated with silver epoxy for connecting to the source and drain electrodes (Figure 8 F–H). In an aquarium containing lemnoideae lema (commonly known as duckweed), the array was evaluated for the real-time detection of K^+^, Na^+^, NH^4+^, NO_3_^–^, SO_4_^2–^, HPO_4_^2–^, and Cl^–^ ions. The ion concentrations in the lemnoideae lema-containing tank were monitored for 3 weeks, revealing a 70–80% drop due to nutrient consumption by the aquatic plant (Figure 8I,J). Another IS-GFET was installed to monitor the outflow of K^+^ ions from living neural cells.^218^ After 2 min of stabilization, the IS-GFET was tested in a glass coverslip (25 nm diameter) containing cultivated U252 human glioma cells, and the K^+^ efflux process from the live cells was measured. The IS-GFET array containing 25 devices was capable of multiplexed detection of K^+^ ions in live cells.

## Biointerfacing and Extracellular Recording by Wearable GFET Devices

Bioelectronic devices capable of capturing and amplifying neural activity signals at soft neuronal tissue interfaces with long-term functionality constitute a promising tool for treating neurological illnesses like epilepsy and Alzheimer’s disease as well as therapeutic uses.^219,220^ New bioelectronic systems are being developed with the promise of successful integration with neural cells. Also, these devices have multimodal functions like sensitive monitoring of neuronal processes, including AP mapping and neural network activation, which have long been desired to understand brain functioning better.^221−223^ Because of its strong resistance against the cell membrane and low resistance between the recording element and the interior of the cells, the patch-clamp microelectrode technology has been widely regarded as a benchmark for precise recording of intracellular activity.^224,225^

Despite substantial advances in the fabrication of large-area, high-density microelectrode arrays, rigid probe-based bioelectronic devices face a significant mechanical and topological mismatch between the electrical probes and cellular networks. Furthermore, the basic structural design of such devices does not allow for scalability for recording large volumetric space such as multiple cells (more than a hundred or thousands of cells) and does not allow for communication across wide and curved cell surfaces without causing cell surface disruption.^226^ As a result, developing new bioelectronic tools with a high spatial and temporal resolution capable of recording intracellular neuronal activities while maintaining device scalability for recording a large network of electrogenic cells are two important goals for advancing *in vitro* or *in vivo* electrophysiology studies.^227−229^ To meet these requirements, researchers are concentrating their efforts on developing highly flexible and stretchable bioelectronics skins as well as mechanically soft as neural cells, through rational device shape, scalability, mechanical qualities, and biochemical variables.^226^

Graphene bioelectronics is a fast-expanding field of research that offers unique prospects for overcoming most of the hurdles and fabricating highly flexible, biocompatible devices for interfacing with biological cells like brain tissues.^55,230^ The unique properties of graphene^231,232^ make it an appealing candidate for fabricating FET arrays capable of achieving stable direct contact with cells and precise electrical recording and amplifying neuronal activity signals. Furthermore, under safe *in vivo* operation settings, GFET arrays may be successfully integrated with biological systems for real-time monitoring of intra- and extracellular phenomena such as cellular excretion and cell membrane potential regulation.^233,234^ The great flexibility of monolayer graphene allows SG-GFETs to be embedded onto ultraflexible and soft substrates, making them appealing for fabricating flexible and soft devices that can be successfully implanted into biological cells without causing cell disruption.^235^ Integrating graphene with neural networks has already been shown to have no effect on neuronal signaling qualities and does not affect nerve cells or tissues.^236^

### Flexible GFET Devices for *In Vitro* Recording of Neuronal Activities

*In vivo* monitoring of neuronal functions has been performed with GFET devices. The use of a GFET device constructed with mechanically exfoliated graphene layers for detecting/recording signals from spontaneously beating embryonic chicken cardiac cells was first reported in 2010.^237^ Recording the extracellular signals was done by monitoring the GFET conductance signal, which was connected to cultivate cardiomyocyte cells. The electrogenic signals were detected by the GFET device with a signal-to-noise ratio >4. This successful integration of a GFET microdevice in living electrogenic cells spurred a succession of theoretical and experimental works to build next-generation wearable microdevices that can capture electrical signals from the nervous system.^238^ The development of flexible and wearable GFET arrays capable of integrating (with a strongly coupled interface) with cell membranes and measuring action potentials (APs) from electrogenic cells is one of the significant advances in this field. Hess et al.^36^ built an array of SG-GFETs to record APs of cardiomyocyte-like HL-1 cells using CVD-grown large-area graphene layers. They cultured the HL-1 cells on the GFET array to form a densely packed layer, and the cell signal was monitored using differential interference contrast (DIC) imaging to demonstrate the presence of a confluent layer of healthy cells. At the same time, they saw a variety of recurring spikes (signals) in the differential current versus time curves for all operational GFET devices, which they attributed to AP propagation across the cells. The data revealed a signal propagation speed of 12–28 m/s and a noise level of 50 V. The use of GFET arrays to record extracellular or intracellular potential signals of neurons is a remarkable breakthrough for current bioelectronic devices.^239^

**Figure 9:**
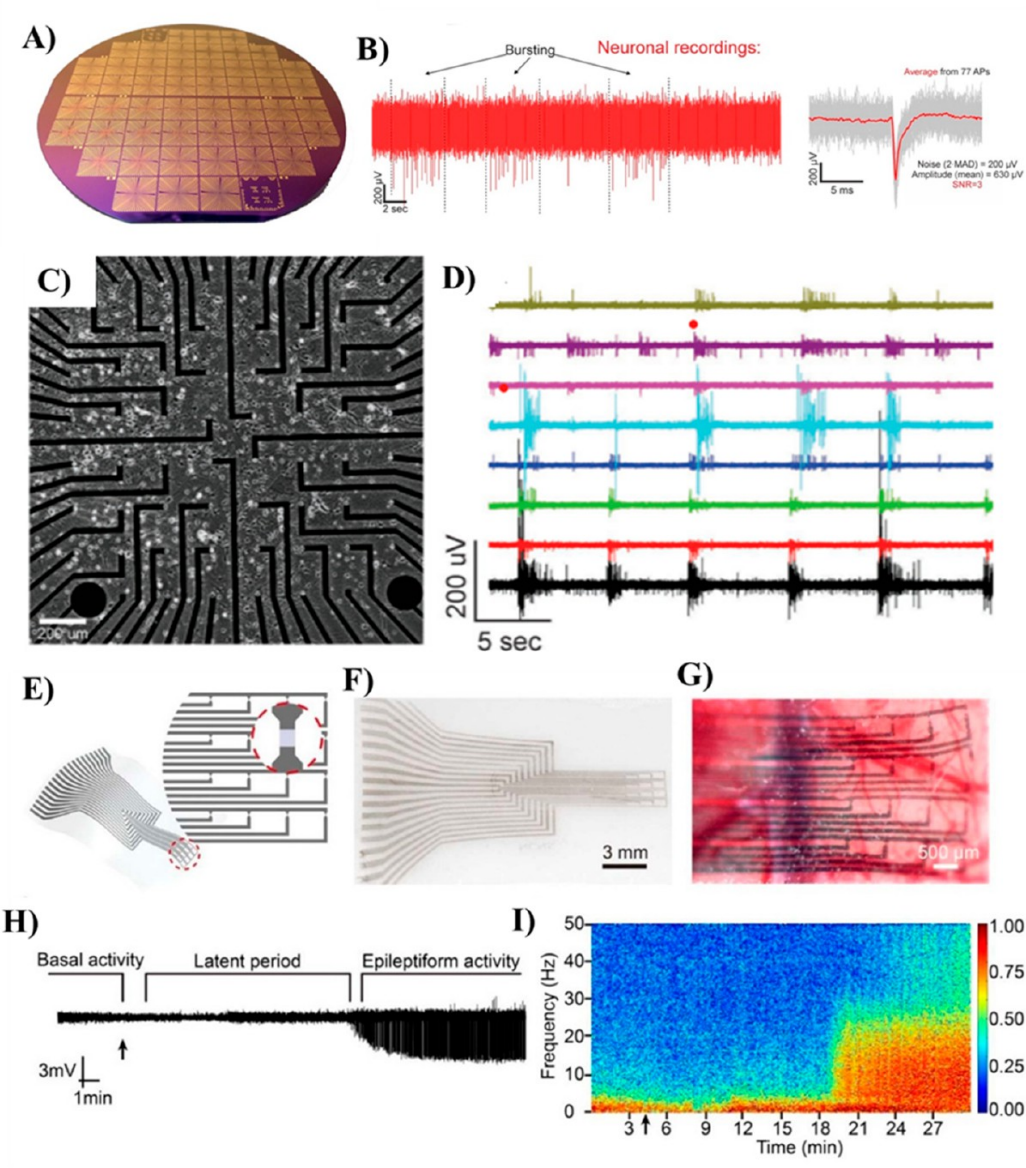
(A) Optical image of design layout of a single GFET chip utilized for *in vitro* recording of neuronal signals. (B) Neuronal recording time tracking features of a burst of intrinsic neuron exhibit alternative periods of bursts at high frequency and spikes at low frequency. (B) An average AP (red) of 77 individual APs (gray). Reprinted by permission from Macmillan Publishers Ltd.: Nature, Kireev, D.; Brambach, M.; Seyock, S.; Maybeck, V.; Fu, W.; Wolfrum, B.; Offenhaüsser, A. *Sci*. *Rep*. **2017**, *7*, 1–12 (ref (54)). Copyright 2017. (C) Microscopic image of neuronal culture grown over a GMEA chip. (D) A timeseries recording of spiking-bursting activity signals on different channels. Reproduced from Graphene Multielectrode Arrays as a Versatile Tool for Extracellular Measurements, Kireev, D.; Seyock, S.; Lewen, J.; Maybeck, V.; Wolfrum, B.; Offenhäusser, A, *Adv*. *Healthc*. *Mater*. Vol. 6, Issue 12 (ref (230)). Copyright 2017 Wiley. (E–I) *In vivo* brain activity recording using highly crumpled all-carbon transistors. (E) Schematic illustration of a 4 × 4 array of an all-carbon GFET device. (F and G) Optical image of the GFET device before and after being placed over the left cortical surface of rat brain. (H) Real-time recording of induced-epilepsy activity using GFET device. The black arrow indicates the time point of penicillin injection. (I) Normalized time-frequency spectral analysis of the time-series data in panel D. (Reproduced from Yang, L.; Zhao, Y.; Xu, W.; Shi, E.; Wei, W.; Li, X.; Cao, A.; Cao, Y.; Fang, Y. Highly Crumpled All-Carbon Transistors for Brain Activity Recording. *Nano Lett*. **2017**, *17* (1), 71–77 (ref (244)). Copyright 2017 American Chemical Society.)

Kireev et al.^54^ used GFETs to capture neural impulses *in vitro* by growing the cortical neural network on a GFET for at least 14 days until it matured (Figure 9A). The bursting activity of the neural network could be recorded after generating action potentials (APs) that propagate through the grown neuronal network. The GFET chip displayed a high signal amplitude (200 V) and a signal-to-noise ratio of about >3 after recording 77 APs (Figure 9B). They also utilized a feedline follower passivation layer, covering the metallic feedlines (drain and source electrodes) and significantly improving cell adhesion at the neurons’ interface with the gate electrode surface. The same authors reported the fabrication of a graphene multielectrode array (GMEA) for *in vitro* recording of APs and spontaneous bursting/spiking neuronal activity in cardiac-like cells and cortical neuronal networks, with a signal-to-noise ratio of 45 ± 22 for HL-1 cells and 48 ± 26 for cortical neuronal networks, respectively (Figure 9C,D).^230^ In this study, they created 64 electrode arrays per chip with dimensions of 1.4 mm × 1.4 mm and then grew rat embryonic cortical neurons for 21–25 days to construct a well-connected neural network with a density of 800 cells mm^2^. The brain activity was collected by real-time monitoring of spiking/bursting activity utilizing GMEA devices. Eight graphene channels in a single device were able to detect high-amplitude spiking/bursting signals (up to 800 V) that occurred every 5–15 s.

Veliev et al.^240^ reported the use of flexible GFET arrays fabricated on transparent and flexible substrates (sapphire, glass, coverslips, and polyimide) for the spontaneous recording of hippocampal neurons APs inside a millimeter-sized PDMS microfluidic chamber. For recording neuronal activity, the hippocampal neurons were cultured over GFET arrays for 21 days to complete electrical maturation. The GFET arrays were initially coated with a synthetic polymer (poly-l-lysin) to promote cell adhesion, which helps to bind the neuron membranes electrostatically with the graphene channel surface. The Dirac point of the GFET device shifted to positive compared to the bare GFET device (i.e., 0.2 V) when cultured live neurons were attached to the graphene channel surface, resulting in a decrease in graphene channel conductance. The negative resting membrane potential of the neural network was primarily responsible for the shift. The arrays could record tiny potential pulses generated by the hippocampal neural network over the GFET surface in different environments with no substantial noise level.

The same authors also developed GFET-arrays of various sizes (*W* × *L* = 1000 × 250 μm^2^, 40 × 250 μm^2^, and 40 × 50 μm^2^) to achieve better transconductance and sensitive recording of ion channel activity inside hippocampus neuron networks.^241^ They did this by growing hippocampus neurons on GFET arrays until they were fully developed electrically (19–21 days *in vitro* (DIV)) with a density of 0.5 × 10^5^ cells/cm^2^. The arrays were coated with poly-l-lysine polymer to improve cell adherence and outgrowth. Ion conduction in the neuron-functionalized GFETs was monitored by measuring the *I*_DS_, keeping the bias voltage (*V*_DS_) and liquid gate potential (*V*_G_) constant. The observed signal perfectly matched the forward and backward ion currents (typically Na^+^ or Ca^2+^) in the membrane channels. Furthermore, through numerical simulation, the authors projected that the inclusion of grain boundaries in the graphene channel would improve electron transmission, ion trapping, and diffusion into the GFET channel, resulting in an increase in the detection response of the GFET device.

Highly promising organ-on-electronic-chip (organ-on-e-chip) based on a three-dimensional (3D) self-rolled graphene biosensor array (3D-SR-Bas) was reported for electrophysiological measurements of human spheroids.^242^ A planner surface of stressed metal/polymer multilayer structure support was designed as a working electrode to fabricate self-rolling arrays of graphene microelectrodes (passive biosensor) and GFET arrays (active biosensor). Upon releasing off from the metal/polymer multilayer surface, it self-rolled into a controlled 3D geometry. The 3D-SR-Bas was employed for recording and mapping electrical signal propagation in stem cells of human cardiomyocyte (CM) spheroids. The device was capable of encapsulating spheroids in direct contact with the biosensor. Twelve microelectrodes (passive biosensor) were arranged in 3D over the CM spheroids and simultaneously recorded the field potentials (FPs). The 3D-SR-Bas could stably record the activities of the CM spheroids with a beating rate of 19 beats per minute. Moreover, adding Ca^2+^ indicator into CM spheroids enabled simultaneous monitoring of Ca^2+^ transients from selected areas. The observed Ca^2+^ spike frequency matched well with the FP spike frequency. Furthermore, the 3D-SR-Bas allowed electrical recording of the individual ionic currents such as Na^+^ current (upstroke), K^+^ current (repolarization), and Ca^2+^ current (plateau phase) across the cell membrane at a single-sensor level with signal-to-noise ratio of ∼9.

### *In Vivo* Recording and Mapping of Cerebral Activities Using GFETs

The creation of bioelectronic devices that can record chronic activity *in vivo*, in sleep, anesthesia, coma, and freely moving animals has been one of the most significant achievements in the field of *in vivo* monitoring of brain activities. For example, GFET devices were conceived and implemented for *in vivo* recording of ultraslow signals from rat brains.^243^ Graphene and carbon nanotubes were used to create a flexible, highly crumpled transistor device. The flexible microelectrode was employed as an electrocorticography (ECoG) probe for *in vivo* recording of epileptic activity in rat brain (Figure 9E–I).^244^ The source–drain electrodes of the all-carbon transistor were designed using porous, CVD-produced CNT film. Following that, the patterned film was transferred to a copper substrate. CVD was then used to incorporate a graphene channel. The rat brain surface was precisely conformed to the produced highly crumpled transistor. The rat brain was then injected with penicillin G sodium to produce epileptic activity, and the population spikes were recorded in real time (Figure 9H). The authors could identify the basel activity, latent period, and epileptiform activity period by recording the population spikes. The latent phase was detected right after the penicillin injection and then vanished within a few minutes.

Blaschke et al.^245^ developed flexible SG-GFET arrays on polyimide substrates with active areas of around 300 μm^2^ (*W* = 20 μm, *L* = 15 μm) for *in vivo* recording of local field potential (LFP) in the brains of sedated rats. The arrays were surgically inserted into the surface of the rat cerebral cortex (using a minimally invasive approach). The devices were highly stable and functional, with a high signal-to-noise ratio of ∼62. For the neural recording using an SG-FET micro transistor, pre-epileptic activity in rat brain was induced by locally injecting bicuculline; the pre-epileptic activity was monitored, and the performance was compared with state-of-the-art Pt electrodes of different sizes (50 and 10 μm). The micro transistor could record averaged interictal spikes greater than the Pt electrodes of two different sizes. Moreover, the transistor could also record a single spike during bicuculline-induced activity with a time-frequency analysis. The key advantage of the fabricated SG-GFET device is that it can be operated at zero bias, avoiding the use of gate voltage, which is promising for *in vivo* recording of essential cellular activities in brain tissues.

Garrido and colleagues investigated SG-GFET-based arrays for *in vivo* recording and mapping of brain electrophysiological signals in rats.^246^ They achieved high-fidelity *in vivo* recording of cortical spreading depression (CSD) signals from rat brains at sub 0.1 Hz frequencies.^246^ The flexible array had a thickness of 12 μm in both epicortical and intracortical designs. Zero insertion force connectors were used to connect the GFET arrays to the recording electronics. The CSD from the rat’s brain was chosen within a wide bandwidth in two craniotomies performed over the left hemisphere of isoflurane-anesthetized wister rats for recording electrophysiological signals using GFET arrays. A bigger craniotomy was made over the primary somatosensory cortex, where the GFET array probe was implanted, and a smaller one was made in the frontal brain, where 5 mM KCl solution was injected to induce CSD. By inducing CSD signals through KCl solution injection, the signals were simultaneously recorded in two frequency bands: a low-pass filtered band (LPF, 0–0.16 Hz) and a high-pass filtered band (BPF, 0.16 Hz to 10 kHz) using a GFET array. The signals detected in the LPF band correspond to a very slow CSD event while the signal in the BPF band was linked to the local potential, indicating that CSD activity is being silenced. The propagation of CSD events was also mapped using a 4 × 4 epicortical GFET array, and the results were compared to high-pass filtered recordings. The CSD event lasted 47 ± 8 s, and the propagation speed was around 8 ± 1 mm/min. Negative shifts of onset signals were seen in the GFET arrays, and certain transistors showed a second negative shift with a larger amplitude than the first. These variations in CSD signals with recovered and remaining depressed brain areas were visible in the mapping but not in traditional microelectrode recording. Moreover, the authors demonstrated the scalability of the SG-GFET array probe consisting of a linear array of 15 SG-GFETs covering the entire depth of a rat cortex. The linear array could record the CSD events in the whole cortex depth, where the transition from long depolarization in the upper layer to hyperpolarization in the deeper layers was clearly recorded. The advancement made in this work highlights the ideal chronic implantable devices for clinical diagnostic tool for understanding brain function and monitoring disease status.

**Figure 10:**
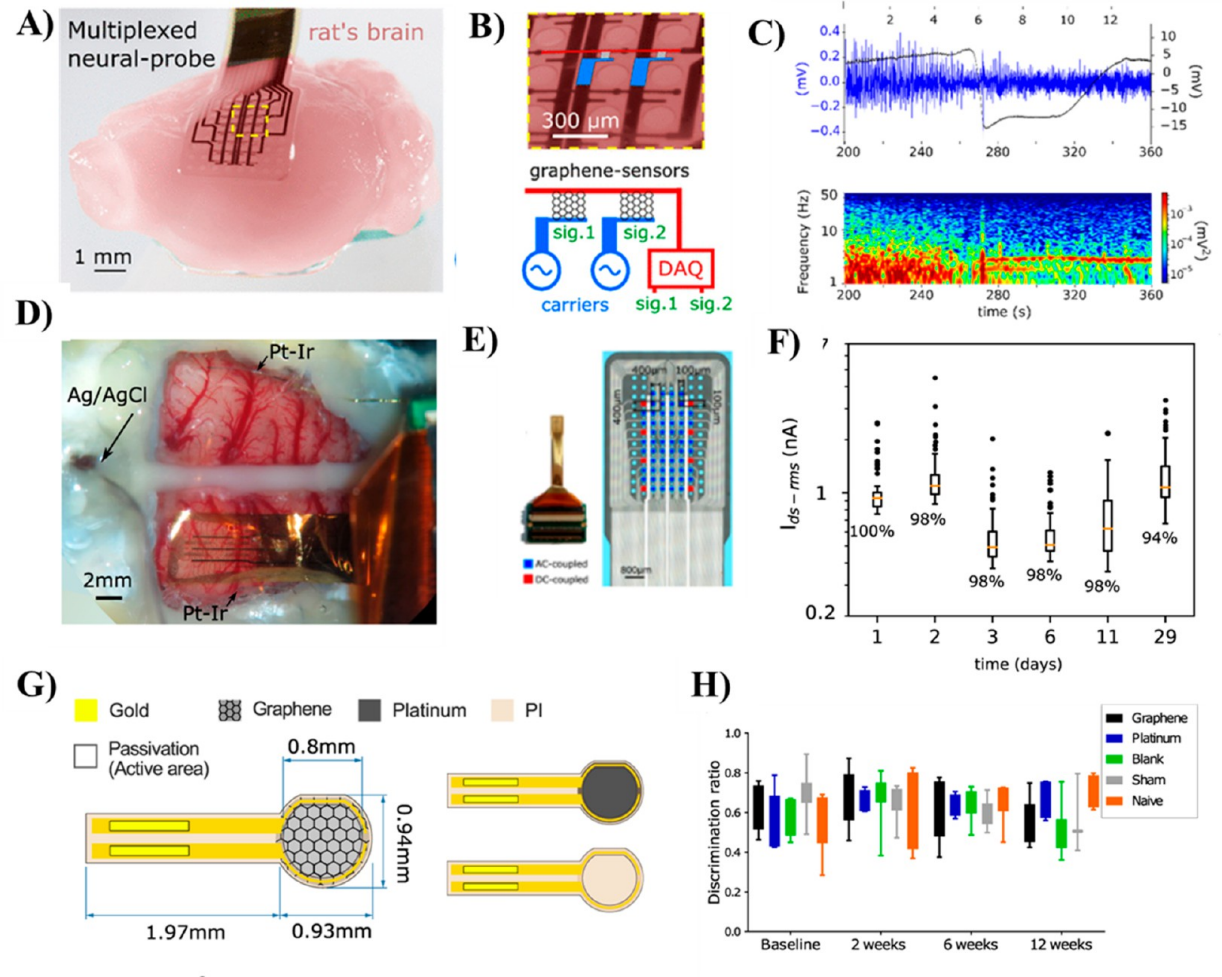
*In vivo* electrophysiological mapping systems. (A–C) Multiplexed GFET-based neural probe for *in vivo* brain mapping. (A) Schematic illustration of flexible 4 × 8 gSGFET arrays. (B) Simplified equivalent circuits of a GFET neural probe. (C) Recording of a typical cortical spreading depression (CSD) event by single SG-GFET. Top: activity in the 1–50 Hz band (blue, left axis) and the wide-band activity (0.001–50 Hz) (black, right axis). Bottom: corresponding spectrogram in the 1–50 Hz band. (Reproduced from Garcia-Cortadella, R.; Schäfer, N.; Cisneros-Fernandez, J.; Ré, L.; Illa, X.; Schwesig, G.; Moya, A.; Santiago, S.; Guirado, G.; Villa, R.; Sirota, A.; Serra-Graells, F.; Garrido, J. A.; Guimerà-Brunet, A. Switchless Multiplexing of Graphene Active Sensor Arrays for Brain Mapping. *Nano Lett*. **2020**, *20* (5), 3528–3537 (ref (45)). Copyright 2020 American Chemical Society). (D–H) GFET-based active sensor arrays for chronic, wireless monitoring of wide frequency band epicortical neural activity. (D) SG-GFET array placed on the rat cortex and (E) photograph of the 64 SG-GGFET arrays mounted on a customized connector (left) and zoomed image of the probe active area (right). (F) Stability response of the SG-GFET array (*n* = 64) for all 64 channels over four week period of implantation. (G and H) Biocompatibility testing of an active SG-GFET array placed in a rat cortex, with schematics of (G) the adopted SG-GFET prototype, and (H) the inflammatory response of the tissue evaluated using ELISA of blood or brain tissue for a panel of inflammatory cytokines over different days after implantation. (Reprinted by permission from Macmillan Publishers Ltd.: Nature, Garcia-Cortadella, R.; Schwesig, G.; Jeschke, C.; Illa, X.; Gray, A. L.; Savage, S.; Stamatidou, E.; Schiessl, I.; Masvidal-Codina, E.; Kostarelos, K.; Guimerà-Brunet, A.; Sirota, A.; Garrido, J. A. *Nat*. *Commun*. **2021**, *12*, 1–17 (ref (247)). Copyright 2021.)

The same research group developed a novel technique incorporating frequency division multiplexing (FDM) of SG-GFETs, which avoids on-site switching and decreases GFET array fabrication complexity for *in vivo* recording of brain events (Figure 10A–C).^45^ The amplitude modulation (AM) by separate carrier signals, which enables the detected multiple brain signals in the active sensor arrays to be communicated across a shared communication channel, is an appealing characteristic of these SG-GFET arrays. The reported SG-GFET array neural probe was successfully validated for *in vivo* recording of wide-band neural activities of a rat’s brain surface. When the neural probe was placed on primary visual cortex V1 (bottom left), it was able to efficiently differentiate distance-dependent signal amplitudes and signal delay. By tracking the propagation of CSD events across the array under anesthetic conditions, the SG-GFET arrays can be utilized for distortion-free recording of infra-low signals of CSD events with high fidelity (Figure 10C).

The same research group used 64-channel GFET-based active sensor arrays for wireless mapping of epicortical brain activity over a wide frequency range from infra-slow to high-gamma frequency bands (Figure 10D,E).^247^ They developed a two-stage trans-impedance amplifier and implemented it in the signal amplification and digitization process to minimize the noise generated by the headstage during the signal amplification and digitization process and the large DC offsets caused by the large dynamic frequency bands. Specifically, the *I*_DS_ current from the SG-GFET was converted into voltage in the first stage, which contains signals of the entire frequency band, including the signals of infra-low-frequency components. In the second stage, they used a high-pass filter to eliminate the DC offsets to convert the analog-to-digital (AD) conversion over the entire scale. The SG-GFET array was used to record epicortical brain activity in freely moving rats for up to 24 h using a wireless recording device, and the 3D motion of the rat was acquired using a motion capture (Mocap) system. The device demonstrated high sensitivity for mapping cortical infra-slow brain activity (ISA, 0.5 Hz) in freely moving animals with high accuracy and spatial resolution over spectral frequencies between 0.015 and 4 Hz with enough signal-to-noise ratio for recording fluctuations in high-frequency LFP dynamics at different time scales. The developed SG-GFET arrays could efficiently record the neural activity with exceptionally high stability (after 6 days of implantation) as shown in Figure 10F. Furthermore, the biocompatibility of the SG-GFET array was accessed using the SG-GFET prototype as displayed in Figure 10G. The developed neural probe showed negligible expression of any cytokine even after 12 weeks of implantation, suggesting adequate biocompatibility and negligible systemic complication caused by the neural probe (Figure 10H). The developed SG-GFET sensor arrays clearly demonstrate that they are highly promising for long-term chronic implantation and long-term and wireless recording of wide frequency band epicortical brain activities.

A flexible array of SG-GFET micro transistors-based depth neural probes (GDNPs) was developed for simultaneous *in vivo* recording of localized full-bandwidth neuronal activity.^248^ The device consisting of 14 recording transistor arrays with an active area of 60 × 60 μm^2^ and a pitch of 100 μm was fabricated on a 10 μm thick polyimide substrate along with two metal levels interconnected by holes. The electrophysiological signals were recorded in awake mice and head-fixed mice by implanting GDNPs in the right hemisphere visual cortex (V1) and lowering them until the tip touched the hippocampus tissue. The probes could reliably record the DC-shifts and spreading depolarizations (SD) associated with seizures in a living rat’s brain at high frequencies with a high spatial resolution. Notably, the GDNPs can stably record the seizure activity for 60 min after injection of drugs. The authors successfully demonstrated the GDNP probes chronically implanted in the right-hemisphere somatosensory cortex rat model of the absence of epilepsy over 10 weeks, and the chronic recording was made 1 to 2 times per week. The GDNP could accurately monitor the seizure activity such as fidelity spontaneous spike-wave discharges and associated infra-low oscillations during the whole implanted period.

## Summary and Future Outlook

In this review, we presented the progress achieved in the fabrication of GFET devices to detect a wide range of biomolecules in a label-free and low-cost manner. Progress on the development of flexible and portable digital GFET biosensors and their sensing capabilities such as sensitivity and selectivity have been assessed. Recent improvements in the design of GFET-based biosensing platforms, together with the use of novel techniques, have enabled ultrasensitive real-time detection of NAs with sensitivity down to aM concentration.^99^ Among the promising strategies adopted so far, the signal amplification approach used by Gao et al.^100^ and the multiplexing of GFET arrays demonstrated by Mensah et al.^106^ stand out. The incorporation of metal NPs into graphene channels improves the electrical conductivity and selectivity of biomolecular conjugation while also increasing the active surface area. All of these factors enhance the performance of GFET-based biosensors.

The development of GFET biosensor devices in portable or wearable chips is highly promising for next-generation point-of-care diagnostics since it provides a simple and versatile way to detect nucleic acids and viral genomes at any location.^2,38,42^ In this regard, adopting CRISPR-Cas technology in GFET biosensor holds promise for the development of effective POC diagnostic tools that do not require signal amplification.^128,129^ Also, integrating these CRISPR-Cas complexes with multiple GFET arrays provides a versatile POC tool for genome-based diagnostics. However, as CRISPR-Cas is a new technology, it must be tested in clinical samples to determine its impact and potential for POC diagnostics.

GFET biosensor-integrated nucleic acid-based assays have remarkable sensitivities that outperform state-of-the-art diagnostics methods like PCR, synthetic biology-based CRISPR, and others and a short diagnosis time of about 80 s.^195^ Thus, the development of portable devices combining CRISPR-Cas technology or nucleic acid assays with GFET devices on paper-based microfluidic devices could be a promising low-cost, next-generation technology for use in biosensor platforms, which offers early detection and diagnosis of cancer biomarkers as well as viral genomes using only a small quantity of liquid samples. Finally, recent advances in the integration of GFET devices with smartphones, commercial electronic chips, and printed circuit boards (PCBs) have made remote detection and real-time monitoring of cancer biomarkers a reality.^42,43^ The advancements made in this area over the last several years have propelled GFET biosensor technology to the next level for use in electronic POC (ePOC) devices for practical applications.

Despite the significant progress made in improving GFET devices and integrating them into microelectronic control and manipulation platforms, various obstacles remain to be overcome before they can be used in commercial applications or testing facilities. The optimal material integration and device structure, the quality of graphene/GO/rGO employed in the conducting channel, device-to-device heterogeneity, biomolecule conjugation techniques, and decreasing Debye screening are some of the difficulties that still need to be addressed before GFET-based biosensors can be commercialized. It is also essential to focus on the underlying issues related to the device’s basic construction and channel material.

Obtaining a single-crystal, high-quality graphene monolayer remains a challenge, even though CVD is considered an effective approach for creating large-area graphene films on Cu foils. Surface flaws and contaminations alter the transport characteristics of graphene channels, changing the features of the graphene channel–biomolecule interface and hence the performance of GFET biosensors. As a result, the development of GFET biosensors relies heavily on the development of efficient techniques for large-scale manufacturing of high-quality single-crystal graphene layers and their transfer to the desired substrates.

Production of GFET biosensors with low device-to-device variation is another challenge that must be overcome not only for large-scale fabrication of these biosensors but also for their reproducible and stable room-temperature operation. In this context, the quality of graphene channels and the techniques for functionalization are two fundamental aspects. Despite significant efforts to create effective functionalization methods, the problems in generating stable (low degradation/deterioration) and high-density surface immobilization of probe molecules or aptamers over the graphene channel remain elusive. Variations in immobilization techniques have a significant impact on GFET biosensor performance and, as a result, device-to-device variability. Therefore, care must be taken in choosing the cross-linking molecules and immobilization procedures, which determine the stability of the channel layer and the density of specific bonding molecules on transistor channels. To solve all of these fundamental issues, continuous research on the design and growth of graphene layers is required with a focus on finding an appropriate deposition process, precision engineering of device properties, and biomolecular conjugation chemistry. Such advancements and breakthroughs would open the way for the development of next-generation detection technologies that might be used for biosensing, diagnosis, healthcare, and environmental monitoring.

In the second part of this review, we presented the progress made on the development of GFET-based bioelectronic devices, which can be used for intra- and/or extracellular electrophysiological recording of action potential from living cells such as cardiomyocyte-like HL-1 cells and neural networks. In comparison to the conventional MEA and patch-clamp approaches, GFET microtransisitors have been created and effectively deployed for *in vivo* recording of cortical brain activities in rats with improved spatial and temporal resolution. The use of GFET arrays to record brain activity has been emphasized. GFET arrays have been used to record cortical spreading depression in rats^246^ and spontaneous pre-epileptic events in the rat brain^249^ with high special-resolution, indicating that future diagnostic tools for monitoring brain processes could be developed with them. However, because of graphene’s unique structure and mechanical properties, GFET microarrays made with graphene as the channel material are not completely compatible with neuronal cells or tissues. This is mostly due to the mechanical mismatch between graphene and brain tissues, which is still a key issue that needs to be addressed for these neural sensors to operate reliably and long-term. Therefore, substantial attention must be paid to the design of channel materials and modulating their mechanical properties in order to ensure that the probe and the probing cells are in close proximity without mechanical stress. In this regard, porous graphene and soft polymer-based injectable meshes have been used to create GFET microarrays. Integration of soft polymers in GFET-based microarray probes appears to be a good solution to address not only the mechanical stress issue but also the probe’s biocompatibility with neuronal cells in order to use them in long-term *in vivo* neuronal activity monitoring. To avoid cell injury and detect ultralow electrophysiological signals over wide areas of neural networks, the GFET array scalability, sophisticated electronic circuits, and signal amplification procedures must be improved. The creation of 3D, extremely flexible, scalable GFET microarrays that can enable ultraflexibility and subcellular feature size could pave the way for significant progress in this field.
